# The relationship between homework time and academic performance among K‐12: A systematic review

**DOI:** 10.1002/cl2.1431

**Published:** 2024-09-18

**Authors:** Liping Guo, Jieyun Li, Zheng Xu, Xiaoling Hu, Chunyan Liu, Xin Xing, Xiuxia Li, Howard White, Kehu Yang

**Affiliations:** ^1^ School of Basic Medical Sciences, Evidence‐based Medicine Centre Lanzhou University Lanzhou Gansu China; ^2^ School of Public Health, Center for Evidence‐based Social Sciences Lanzhou University Lanzhou Gansu China; ^3^ Laboratory of Evidence Based Medicine and Knowledge Translation of Gansu Province Lanzhou Gansu China; ^4^ School of Basic Medicine Shanghai University of Traditional Chinese Medicine Shanghai China; ^5^ Institute of Higher Education Lanzhou University Lanzhou Gansu China

**Keywords:** academic performance, homework time, K‐12, systematic review

## Abstract

**Background:**

Homework is a common educational task given to students around the world. It demands mental exertion, but staying focused can be challenging, especially for K‐12 students. Too much homework can increase their cognitive load and mental fatigue, leading to decreased motivation and performance. This can cause boredom with homework and learning. To lessen their load and make homework more effective, it is important to establish the connection between homework duration and academic achievement.

**Objectives:**

To evaluate the relationship between homework time and academic performance among K‐12 students.

**Search Methods:**

On November 5, 2021, we retrieved articles from a variety sources. Firstly, we searched 10 electronic databases for related publications, including Academic Search Premier, APA PsycArticles, APA PsycInfo, Business Source Premier, Education Resources Information Center (ERIC), Journal Storage (JSTOR), Learning and Technology Library (LearnTechLib), OCLC FirstSearch, Social Sciences Citation Index (Web of Science), and Teacher Reference Center. We also searched two publisher platforms: ScienceDirect and Taylor & Francis Online Database. Secondly, we consulted five educational organization website such as, American Educational Research Association, Best Evidence Encyclopedia, Education Endowment Foundation, European Educational Research Association, What Works Clearinghouse, and the Open Grey database for unpublished studies. We then searched Open Dissertations and ProQuest Dissertations & Theses Global databases to locate the relevant dissertations and theses. Additionally, we hand‐searched seven educational journals to identify unpublished documents, reports, and potential studies not indexed in the databases. Lastly, we searched Campbell Library to identify relevant reviews and primary (and nearly eligible studies) in these reviews. We also searched Google Scholar for related studies and checked the citations of eligible studies as well as their bibliographies.

**Selection Criteria:**

Studies with the following criteria were included:

− *Population:* K‐12 school students with no disabilities or not attending special education schools;
− *Intervention:* Homework assigned regularly by schoolteachers to students to complete during non‐school hours;
− *Comparison:* Different time spent on the homework;
− *Outcomes:* Academic performance was the primary outcome. The secondary outcomes were academic motivation and the quality of homework;
− *Study design:* Treatment‐control group design or comparison group design studies.

**Data Collection and Analysis:**

We reviewed the titles, abstracts, and full texts of the retrieved records. Our team extracted and coded all relevant information from the studies that met our inclusion criteria. To evaluate the risk of bias, we used the Cochrane Risk of Bias tool for randomized controlled trials and ROBINS‐I for non‐randomized controlled trials. A random‐effect meta‐analysis was conducted to determine the effect of homework on academic achievement as compared to no homework. A funnel plot, trim‐and‐fill method and Egger's test were used to test for any publication bias. Due to the insufficient data on homework duration and academic achievement, we analyzed these data using qualitative synthesis.

**Main Results:**

Eleven publications were identified that examined the relationship between homework duration and academic outcomes using an experimental design. Based on their focus, we categorized them into two groups: comparisons of homework with no homework and comparison of homework with less homework. There were 10 articles with 14 independent reports that compared academic performance between students who did homework and those who did not. Overall, the meta‐analysis revealed that the students who did homework had better academic performance than that those who did not (*n* = 14; *g* = 0.45, 95% confidence interval [CI]: 0.24–0.66; *Q* = 454.30, *I* ^2^ = 71.30%, *τ* ^2^ = 0.11), especially in arithmetic computation (*n* = 5; *g* = 0.46, 95% CI: 0.17–0.75; *Q* = 13.03, *I* ^2^ = 69.29%, *τ* ^2^ = 0.07) and arithmetic problems solving (*n* = 6; *g* = 0.17, 95% CI: 0.02–0.33; *Q* = 6.87, *I* ^2^ = 27.17%, *τ* ^2^ = 0.01), but not in arithmetic concepts (*n* = 3, *g* = −0.02, 95% CI: −0.22–0.18; *Q* = 1.46, *I* ^2^ = 0.00%, *τ* ^2^ = 0.00). Two experiments explored the effectiveness of homework moderated by homework time. In Koch (1965), the effects of long daily homework (20–30 min) and short daily homework (10–15 min) were compared. The authors found that achievement in arithmetic concepts was higher with long homework assignments every day. Recently, Dolean and Lervag (2021) confirmed the effect of homework on writing skills, and their findings were consistent with those of Koch (1965), who found that increasing time spent on homework was associated with greater writing achievement (average 20 min each time).

**Authors' Conclusions:**

Homework could be used as a supplement to enhance the academic performance of primary school students. However, the optimal amount of time they should dedicate each day to homework to achieve the best results remains uncertain. More high‐quality experiments are needed to determine the ideal homework duration for these students. Furthermore, additional research is required to understand the impact of homework on secondary school students.

## 1 PLAIN LANGUAGE SUMMARY

### 1.1 Limited experimental studies of the optimum time of homework among K‐12

The available studies suggested large effects of homework on academic performance among primary school students, but few studies evaluate the optimum time spent on homework to improve academic achievement for K‐12 students.

### 1.2 What is this review about?

Homework is a common activity among K‐12 students, and numerous observational meta‐analyses have confirmed the positive relationship between homework and academic performance. However, considering children's limited energy and attention, several researchers have proposed an optimum time to spend on homework.

This Campbell review examines the effects of homework on academic performance among K‐12 students with experimental studies and estimates the optimum time that should be spent on it.

### 1.3 What studies are included?

This review included eleven experimental studies, eight of them evaluated the effects of homework for K‐12 students and two studies estimated the time effect of homework. Only one study was published online recently, in 2021, and the remaining studies spanned the period from 1939 to 1995.

### 1.4 What is the relationship between homework and academic performance?

Homework has a beneficial effect on primary school student's academic achievement, especially in improving their arithmetic computational skill and arithmetic problem‐solving skills. However, the optimum duration spent on homework is not clear.

### 1.5 What do the findings of this review mean?

Homework is recommended as a supplement to improve academic performance in primary school students. However, scarce and outdated empirical studies included in this review suggest an urgent need for high‐quality experiments to explore the optimal duration to make full use of homework.

### 1.6 How up‐to‐date is this review?

The review authors searched for studies up to November 5, 2021.

## 2 BACKGROUND

### 2.1 Description of the condition

Homework is defined as “any task assigned by schoolteachers intended for students to carry out during non‐school hours” (Cooper, 1989). This definition explicitly excludes (a) in‐school guided study; (b) home study courses delivered through the mail, television, audio or videocassette, or the internet; and (c) extracurricular activities such as sports and participation in clubs (Cooper et al., 2006). Nowadays, thanks to technology, web‐based homework has become increasingly popular among teachers. Platforms like Google Classroom,^1^ Firefly,^2^ eSchools,^3^ and Moodle^4^ (Mendicino et al., 2009) allow students to complete their homework online and teachers to provide immediate feedback (Callahan, 2016; Lucas, 2012; Mendicino et al., 2009). Therefore, in this systematic review, homework also includes online tasks performed outside school.

The purpose of homework can be divided into instructional and non‐instructional objectives (Lee & Pruitt, 1979). The most common instructional purpose of homework includes review, preview, and extension (Becker & Epstein, 1982; Lee & Pruitt, 1979; Muhlenbruck et al., 1999). Review assignments allow students to practise newly acquired skills or review material learned in class. Preview assignments introduce new skills or materials before the class to help students prepare for unfamiliar knowledge (Muhlenbruck et al., 1999). Extension assignments involve transferring previously learned skills to new situations (Cooper et al., 2006; Lee & Pruitt, 1979). The non‐instructional purpose of homework varies, including forming better study habits, increasing students' sense of responsibility, enhancing awareness of independent learning, and building communication among parents, children, and teachers (Becker & Epstein, 1982; González et al., 2001; Lee & Pruitt, 1979; Muhlenbruck et al., 1999; Van Voorhis, 2003). However, homework can also be used as a form of punishment (Epstein & Van Voorhis, 2001).

Students worldwide are frequently assigned homework as part of their educational activities, which is considered a hallmark of the educational excellence movement. Homework policies vary globally. In China, Primary 1 and 2 students are not assigned any homework, and homework for Primary 3 to 6 students is limited to maximum of 60 min, while junior high school students are allowed up to 90 min per day (Ministry of Education of the People's Republic of China, 2021). In Singapore, the general guideline for time spent on homework is about 30 min to an hour for Primary 1–2 students, 1–1.5 h for Primary 3 and 4 students and 1.5–2 h for Primary 5 and 6 students (South View Primary School, 2024). The UK had specific mandates for homework ranging from no homework for kindergarteners to a maximum of 2.5 h per day for Years 10 and 11 (Cooper & Nye, 1994), but these guidelines were scrapped in 2012 and autonomy was given to headteachers and school leaders (Frog Education, 2019). Similarly, in the 1990s, 35% of US districts explicitly stipulated homework frequency and duration, with recommended daily averages of 40 min for primary school students, 70 min for junior high school students, and 100 min for senior high school students (Roderique et al., 1994). Recently, some US schools have adopted no‐homework policies in response to the Common Core curriculum (Mae Gambong Luengas & Deloy, 2022). Finland, on the other hand, has no national homework policy, and Finnish students have significantly less homework than other nations, with an average of less than 30 min per day (Federick, 2020).

Initially, the level of homework was widely accepted by parents. However, as the volume of homework increased, parents and scholars began to realize the burden it placed on students. Parents complained that children lost their childhood and called for less homework (Gill & Schlossman, 2003). Similarly, in the mid‐19th century, homework became commonplace in the United Kingdom, and its increased level became a topic of much debate in the 1880s in response to the introduction of payment according to results and other factors for teachers (Hallam, 2004). In 2016, the World Health Organization (WHO) reported that students feel pressured from schoolwork (World Health Organization Regional Office for Europe, 2016). Meanwhile, parents continued to express concerns about excessive homework assigned to their children (Gill & Schlossman, 2003, 2004; Jerrim et al., 2019; Xue & Zhang, 2019).

The topic of whether or not homework improves academic performance has been a subject of debate for over a century (Cheema & Sheridan, 2015; Cooper et al., 2006; Cooper, 1989; Kitsantas et al., 2011; Kralovec & Buell, 2000; Trautwein & Lüdtke, 2007). Although several meta‐analyses of the relationship between homework and performance have shown a positive correlation between homework time and academic performance (Baş et al., 2017; Cooper et al., 2006; Cooper, 1989; Fan et al., 2017), it is challenging to establish causality. It is possible that more academically inclined students who typically score better grades complete their homework more thoroughly and conscientiously. On the other hand, students who struggle academically may put in more effort to catch up on their studies at home.

Regardless, it is possible that the effects of homework are not linear. Some evidence suggests that increasing homework duration can improve academic performance, but there is a point where too much homework can actually lead to a decline in performance (Ackerman et al., 2011; Krejtz et al., 2018; Reteig et al., 2019). For example, based on data from the National Center for Education Statistics (NCES) survey of 58,000 high school students in grades one and two, Keith (1982) found that increasing the amount of homework can improve performance for individuals at any level of ability. However, there is a limit to how much homework can be beneficial, and exceeding this limit would lead to a decline in performance. Homework can play a compensatory role, but it must be increased moderately and not beyond a certain duration (Keith, 1982).

### 2.2 Description of the intervention

The intervention we consider in this review is homework assigned by schoolteachers to students during their non‐school hours, without additional teaching or support such as activities in a study club. We excluded flipped learning because the homework in flipped classrooms typically consists of instructional videos, rather than problem sets without additional instruction; Moreover, the students benefit from traditional homework as it extends the school day, while the homework in a flipped classroom is still considered part of teaching (Blazer, 2009; de Araujo et al., 2017). The comparison condition was different amounts of time spent on the homework; we divided it into groups of 0–15, 16–30, 31–45, 46–60min, 61–90, 90–120 min, and more than 120 min. Any type of homework was included, like written, oral, or practical homework. We excluded homework sources like parents or teachers from extracurricular activities such as sports and participation in clubs, as well as in‐school guided study and home study courses. We also excluded homework related to psychotherapy.

### 2.3 How the intervention might work

**Figure 1:**
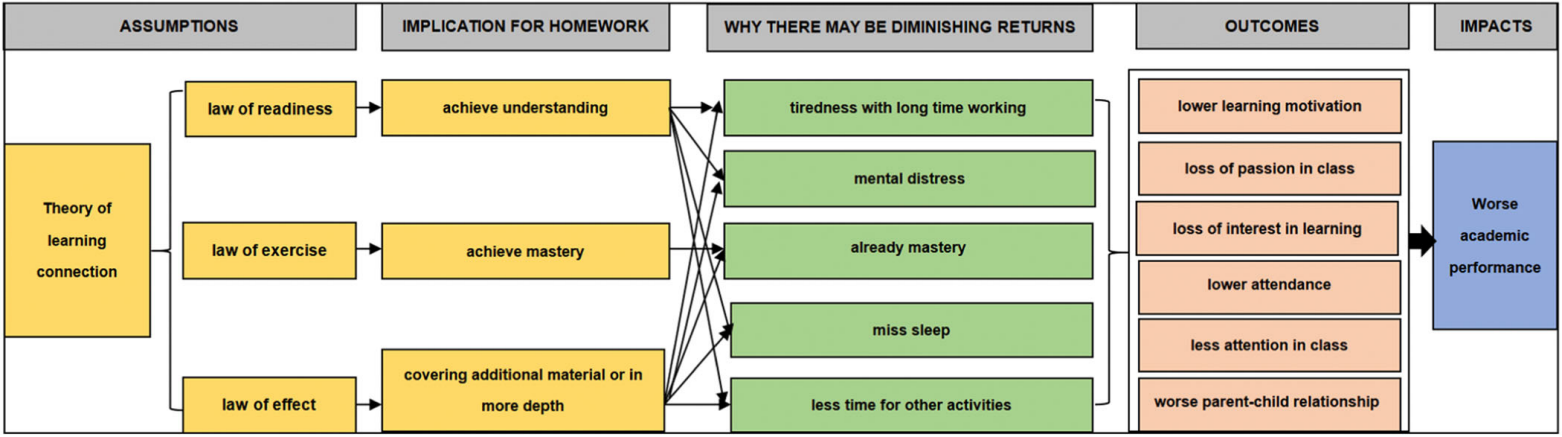
Conceptual framework for the intervention and outcomes of homework.

The conceptual framework for this review was the theory of change, which describes how homework may affect academic performance. Figure 1 provides a visual representation of the framework, which shows how interventions are hypothesized to lead to the intended outcomes.

As in Figure 1, the law of readiness suggests that before commencing a certain learning activity, if the learners perform well in the preparatory stages (including physiological and psychological performance) related to the corresponding learning activities, they can understand the learning content more rapidly (Muhammad, 2015). Second, the law of exercise suggests that repetition of relevant actions in practice will enhance learned connections. Third, the law of effect indicates that all types of positive or negative feedback that learners receive while learning will strengthen or weaken the cognitive connections that they have formed.

However, excessive homework can lead to cognitive overload and mental fatigue, which may reduce students' readiness for learning. This can result in tiredness and anxiety, leading to decreased performance and inadequate feedback (Ackerman et al., 2011; Krejtz et al., 2018; Reteig et al., 2019). Moreover, if students spend excessive time on homework, they may lose time to participate in other activities that could contribute to their overall development.

It is also essential to recognize that spending more time on homework does not always lead to better academic performance. Once students have fully understood the homework content, completing additional homework may not be beneficial. Rather, there may be diminishing returns on the time spent on homework, which can eventually reach zero (Ackerman et al., 2011; Bartelet et al., 2016). Additionally, if students miss out on sleep due to homework, they may experience reduced performance in class or tests, further undermining their learning readiness (Reteig et al., 2019).

### 2.4 Why it is important to do this review

#### 2.4.1 Previous reviews

Several systematic reviews have explored the effectiveness of homework in improving students' performance. However, they assumed a linear relationship between the time spent on homework and performance, without considering how homework duration affects students' autonomous motivation (Bartelet et al., 2016). Even a summary of the evidence by Hallam (2004) did not suggest how much time students should spend on homework (Hallam, 2004). The UK Education Endowment Foundation's (EEF) toolkit entry for secondary school homework emphasized the importance of quality over quantity, but quantity still is a key factor that affects its quality. Therefore, extant reviews left the practical question of homework duration unanswered.

In 1989, Cooper conducted a review of the relationship between homework and performance. The results showed that the average correlation for students in primary‐, middle‐, and high‐school between the amount of homework and performance was zero; for students in middle school, the correlation was 0.07, whereas for high school students, the correlation was 0.25 (Cooper, 1989). In 2006, Cooper et al. (2006) conducted another systematic review of the effectiveness of homework at improving academic performance. The results showed that the correlation between homework time and performance for high‐school students was 0.25, but for middle‐school students, it was nearly zero. However, they did not explicitly consider homework time (Cooper et al., 2006). Fan et al.(2017) conducted a meta‐analysis of the studies published between 1986 and 2015, and demonstrated a small but positive relationship between homework duration and math/science attainment, with a correlation of 0.145 (Fan et al., 2017).

All studies above assumed that more homework always leads to better performance, with a linear correlation between the two. However, it is important to consider that homework can be tedious and mentally taxing, and there are limits to one's ability to concentrate. To maximize the effectiveness of homework, it is important for teachers, school administrators, parents, and students to determine the optimal amount of time to spend on homework. In previous studies, the correlation coefficient was used as a measure of the strength of the linear relationship between homework and performance.

#### 2.4.2 The contribution of this review

Homework can be a valuable tool for improving students' academic performance, whether it is for preparation, practice, extension, or application. Previous reviews have shown that homework is effective in this regard. However, it is important to note that more homework does not necessarily equate to better results. The amount of homework assigned should be limited by students' ability to concentrate for extended periods. This systematic review categorizes participants into groups based on the amount of time spent on homework (0–30, 31–60, 61–90, 90–120 min, and more than 120 min) and compares their test scores to identify the relationship between homework time and academic performance. This information will be useful for teachers and parents to better understand the importance of homework and will provide guidance for teachers in assigning appropriate amounts of homework.

## 3 OBJECTIVES

This review synthesized the results from publications focused on homework duration and academic performance and assessed the relationship between the two factors. Our objectives were the following:

1. To identify the extent of the relationship between homework time and students' academic performance;
2. To analyze differences in the effectiveness of homework time across genders, grades, subjects, and regions; and
3. To identify the potential factors that affect homework time, such as academic subject, task difficulty, type of homework, mode of homework, parental involvement, and feedback on homework.

## 4 METHODS

### 4.1 Criteria for considering studies for this review

#### 4.1.1 Types of studies

According to our protocol (Guo et al., 2021), we included treatment‐control group designs or comparison group designs, namely randomized controlled trials (RCTs) and non‐randomized controlled trials (NRCTs), such as cohort studies, controlled pre‐ and post‐treatment studies, and interrupted time‐series studies. To be included, studies needed to explore the effect of homework duration on academic performance by comparing test score differences between different groups before and after the intervention, and explicitly report the time spent on homework, as well as the mean and standard deviation of academic achievement. Due to the language restrictions within our team, we included only studies published in English.

#### 4.1.2 Types of participants

This review focused on K‐12 school students, excluding those with disabilities and those attending special education schools. If a primary study included a mixed sample of students, we only considered the sub‐sample without special needs students, if that information was provided. This is because the effects and context may differ for individuals with disabilities.

#### 4.1.3 Types of interventions

We explored the relationship between homework duration and academic performance by comparing academic scores obtained given different homework duration. The eligible intervention studies needed to explicitly state that the intervention was regular homework assigned by schoolteachers to students to complete during non‐school hours, with the aim of improving academic achievement, regardless of the nature of the homework content. Furthermore, we included only school‐based interventions; homework was excluded if allocated by other people, such as parents or teachers from extra‐curricular schools, or study clubs, or was associated with extracurricular activities such as sports and participation in clubs. Homework related to psychotherapy was also excluded. The comparison condition was the different duration of homework.

#### 4.1.4 Types of outcome measures

The objective of the review was to explore the impact of homework on students' academic outcomes. We extracted homework time and academic performance from the studies. Homework duration was an exact time or a time frame reported by students or parents. Academic performance was measured by a teacher, exam results, and/or by the research team using any valid standardized test.

Valid standardized tests were considered norm‐referenced tests (e.g., Gates‐MacGinitie Reading Tests and Star Math), state‐wide tests (e.g., Iowa Test of Basic Skills), and national tests (e.g., National Assessment of Educational Progress). If the nature of the test was not clear from the description of outcome measures in the studies, we used electronic sources to determine whether the test was standardized.

The outcome was academic performance (test score and standard deviation); studies that measured academic performance and homework time were included.

#### 4.1.5 Duration of follow‐up

There is no restriction on the duration of follow‐up.

#### 4.1.6 Types of settings

Only the studies conducted in K‐12 schools were included.

### 4.2 Search methods for identification of studies

#### 4.2.1 Electronic searches

The following databases were searched without language limits on November 5, 2021:

- Academic Search Premier (via EBSCOhost)
- APA PsycArticles (via EBSCOhost)
- APA PsycInfo (via EBSCOhost)
- Business Source Premier (via EBSCOhost)
- ERIC (https://eric.ed.gov/)
- JSTOR (https://www.jstor.org/)
- LearnTechLib (https://www.learntechlib.org/)
- OCLC FirstSearch (https://firstsearch.oclc.org/)
- Social Sciences Citation Index (Web of Science, Clarivate)
- Teacher Reference Center (via EBSCOhost)

The following Publisher platforms were searched on November 5, 2021:

- ScienceDirect (https://www.sciencedirect.com/)
- Taylor & Francis Online Database (https://www.tandfonline.com/)

All the search strategies were developed under the supervision of Kehu Yang, the honored chairman of the National Medical Literature Information Education (MLIRE) in China, who has rich experience of information retrieval. The search strategy for each database/platform is provided in Table 1.

**Table 1:** The search strategy and results for each electronic database/platform.

Source	Search strategy	Records
*Electric databases*		
Academic Search Premier (via EBSCOhost)	#1 TI (“homework” OR “home‐work”) OR SU (“homework” OR “home‐work”)#2 AB “achievement” OR “performance” OR “grade” OR “score” OR “academic achievement” OR “gpa” OR “academic performance”#3 AB “k‐12” OR “preschool student” OR “pre‐school student” OR “kindergarten student” OR “middle school student” OR “high school student” OR “senior school student” OR “primary school student” OR “pupil” OR “schoolchild” OR “junior high school student” OR “school‐age”#4 #1 AND #2 AND #3	382^a^
APA PsycArticles (via EBSCOhost)		
APA PsycInfo (via EBSCOhost)		
Business Source Premier (via EBSCOhost)		
Teacher Reference Center (via EBSCOhost)		
ERCI https://eric.ed.gov/	(title: “homework” OR “home‐work”) AND (abstract:“k‐12” OR “preschool student” OR “pre‐school student” OR “kindergarten student” OR “middle school student” OR “high school student” OR “senior school student” OR “primary school student” OR “pupil” OR “schoolchild” OR “junior high school student” OR “school‐age”) AND (abstract: “achievement” OR “performance” OR “grade” OR “score” OR “academic achievement” OR “gpa” OR “academic performance”)	2319
JSTOR https://www.jstor.org/	(ti:(“homework” OR “home‐work”) AND ab:(“achievement” OR “performance” OR “grade” OR “score” OR “academic achievement” OR “gpa” OR “academic performance”))	59
OCLC FirstSearch https://firstsearch.oclc.org/	(su: homework OR su: home‐work) and (su: K‐12 OR su: preschool w student* OR su: pre‐school w student* OR su: Kindergarten w student* OR su: middle w school w student* OR su: high w school w student* OR su: senior w school w student* OR su: primary w school w student* OR su: pupil OR su: schoolchild OR su: junior w high w school w student* OR su: school‐age) and (su: achievement OR su: performance OR su: grade OR su: score OR su: academic w achievement* OR su: GPA OR su: academic w performance)	104
LearnTechLib https://www.learntechlib.org/	title: homework	164
Social Sciences Citation Index (Web of Science, Clarivate)	#1 TI=homework OR AB=homework#2 TI=home‐work OR AB=home‐work#3 #1 OR #2#4 TS = K‐12 OR TS=preschool student* OR TS=pre‐school student* OR TS=Kindergarten student* OR TS= middle school student* OR TS=high school student* OR TS=senior school student OR TS=primary school student* OR TS=pupil OR TS= schoolchild OR TS= junior high school student* OR TS=school‐age#5 TS=achievement OR TS=performance OR TS=grade OR TS=score OR TS= academic achievement* OR TS = GPA OR TS=academic performance#6 #3 AND #4 AND #5	1832
*Publisher platforms*		
ScienceDirect https://www.sciencedirect.com	#1 TI=homework OR AB=homework#2 TI=home‐work OR AB=home‐work#3 #1 OR #2#4 TS = K‐12 OR TS=preschool student* OR TS=pre‐school student* OR TS=Kindergarten student* OR TS= middle school student* OR TS=high school student* OR TS=senior school student* OR TS=primary school student* OR TS=pupil OR TS= schoolchild OR TS= junior high school student* OR TS=school‐age#5 TS=achievement OR TS=performance OR TS=grade OR TS=score OR TS= academic achievement* OR TS = GPA OR TS=academic performance#6 #3 AND #4 AND #5	555
Taylor & Francis Online Database https://www.tandfonline.com	[[Publication Title: homework] OR [Publication Title: home‐work] OR [Publication Title: homework] OR [Publication Title: home‐work]] AND [[Abstract: k‐12] OR [Abstract: “preschool student”] OR [Abstract: “pre‐school student”] OR [Abstract: “kindergarten student”] OR [Abstract: “middle school student”] OR [Abstract: “high school student”] OR [Abstract: “senior school student”] OR [Abstract: “primary school student”] OR [Abstract: pupil] OR [Abstract: schoolchild] OR [Abstract: “junior high school student”] OR [Abstract: “school‐age”]] AND [[Abstract: achievement] OR [Abstract: performance] OR [Abstract: grade] OR [Abstract: score] OR [Abstract: “academic achievement”] OR [Abstract: gpa] OR [Abstract: “academic performance”]]	17
*Dissertations and theses*		
OpenDissertations (via EBSCOhost)	#1 TI (“homework” OR “home‐work”) OR SU (“homework” OR “home‐work”)#2 AB “achievement” OR “performance” OR “grade” OR “score” OR “academic achievement” OR “gpa” OR “academic performance”#3 AB “k‐12” OR “preschool student” OR “pre‐school student” OR “kindergarten student” OR “middle school student” OR “high school student” OR “senior school student” OR “primary school student” OR “pupil” OR “schoolchild” OR “junior high school student” OR “school‐age”#4 #1 AND #2 AND #3	‐
ProQuest Dissertations & Theses Global https://www.proquest.com	(ab(“k‐12” OR “preschool student” OR “pre‐school student” OR “kindergarten student” OR “middle school student” OR “high school student” OR “senior school student” OR “primary school student” OR “pupil” OR “schoolchild” OR “junior high school student” OR “school‐age”) AND ab(“achievement” OR “performance” OR “grade” OR “score” OR “academic achievement” OR “gpa” OR “academic performance”)) AND (su(“homework” OR “home‐work”) OR ti(“homework” OR “home‐work”))	
Total		5179

^a^ This number includes the records from the databases of Academic Search Premier, Business Source Premier, Teacher Reference Center, APA PsycArticles, APA PsycInfo (via EBSCOhost) and OpenDissertations, which were searched via EBSCOhost (http://search.ebscohost.com/) using the same search strategy.

Compared with protocol, we conducted an extra search of the LearnTechLib on November 5, 2021.

#### 4.2.2 Searching other resources

##### Unpublished studies

We searched the following sources to identify relevant unpublished studies and reports using the keyword “homework.” Searches were conducted up to November 5, 2021.

- American Educational Research Association (http://www.aera.net/)
- Best Evidence Encyclopedia (https://bestevidence.org/)
- Education Endowment Foundation (https://educationendowmentfoundation.org.uk/)
- European Educational Research Association (http://www.eera-ecer.de/)
- Open Grey (http://www.opengrey.eu/)^5^
- What Works Clearinghouse (https://ies.ed.gov/ncee/wwc/)

##### Dissertations and theses

We searched OpenDissertations (via EBSCOhost) and ProQuest Dissertations & Theses Global databases for dissertations on November 5, 2021. The search strategies are shown in Table 1.

##### Hand searching

Based on the scope of journals and their 5‐year impact factors, we selected the following international journals and hand‐searched them for relevant studies on November 5, 2021.

- *American Educational Research Journal*
- *Educational Psychologist*
- *Learning and Instruction*
- *Journal of Educational Research*
- *Journal of Educational Psychology*
- *Journal of Research on Educational Effectiveness*
- *Journal of Experimental Education*

##### Citation searching

We searched the Campbell Library (https://www.campbellcollaboration.org/better-evidence.html) to identify previous systematic reviews related to homework, and then scanned the primary studies included (and nearly eligible studies listed in excluded section) in these reviews.

We also searched Google Scholar using the keyword “homework” on November 5, 2021, and all the titles of results and abstracts were screened and evaluated based on our inclusion and exclusion criteria. We stopped the search when there were five consecutive pages with no relevant studies. Additionally, we checked the forward citations of eligible studies as well as their bibliographies.

### 4.3 Data collection and analysis

#### 4.3.1 Selection of studies

The selection of studies was performed independently by the first two reviewers (Guo LP and Jieyun Li) in Rayyan (https://rayyan.qcri.org/). All titles and abstracts of the records identified after retrieval were screened, the potentially relevant references were retrieved in full‐text, and the primary studies that met our criteria were included for further data extraction. Any discrepancies between the reviewers were resolved by consensus with another reviewer (Kehu Yang). The study screening process was based on the PRISMA guidelines (Moher et al., 2009).

#### 4.3.2 Data extraction and management

According to our protocol (Guo et al., 2021), the information extraction and coding form consisted of two parts. The first was general information, including the information of primary study (publication source, year of publication, and year of data collection), sample characteristics (e.g., sample size, gender, grade level, region, family economic status, parental education level), methodological characteristics (e.g., sampling method, measures of homework duration, and measures of academic performance), and intervention characteristics (e.g., subject, mode of homework, and type of homework). The other was the effect size, including that of homework duration and test score. Before the formal data extraction, we performed three rounds of extraction with a pre‐piloted data extraction form. This process was conducted independently by two of the authors (Zheng Xu and Xing Xin). Disagreements between coders were resolved by discussions with another author (Xiuxia Li).

− If a study contained multiple interventions (e.g., different homework modalities such as online vs. book‐based), eligible data were extracted by the reviewers;
− For academic performance, means, standard deviations (or information by which to estimate standard deviations), and the number of participants in each group were extracted. If more than one measure was reported, we extracted all measures and planned to analyze the measurement method as a moderator.
− We extracted the homework duration reported in the primary studies and coded the data as presented, either as categorical or continuous data. We created a data set of continuous variables, using the mid‐point for data reported in categorical form, and at least two categorical data sets. The multiple categorical data sets were used to test sensitivity for the chosen thresholds; the mean and standard deviations were calculated in each group. If the weekly homework duration was reported instead of the daily duration, we divided the total homework duration by five. If the homework duration was listed in hours, we converted it to minutes.
− For controlled pre‐ and post‐intervention studies, mean or median change from baseline scores were extracted. If change scores were unavailable or could not be computed, post‐intervention values were extracted by the reviewers.

#### 4.3.3 Assessment of risk of bias in included studies

For randomized controlled trials, the Cochrane Risk of Bias tool 2.0 (ROB 2) was used to assess the quality of the method and potential limitations (Higgins & Green, 2011). For non‐randomized studies (including cohort studies, controlled pre‐ and post‐intervention studies, and interrupted time‐series studies), the risk of bias in non‐randomized studies of interventions (ROBINS‐I) was used to check the quality of the individual study (Sterne et al., 2016). The risk of bias assessment was conducted by two of the authors (Zheng Xu and Xing Xin). Any disagreement was resolved by discussion with another author (Xiuxia Li). The results of the ROB analysis will be used to assess the credibility of the review findings, and as a moderator in the analysis of heterogeneity.

#### 4.3.4 Measures of treatment effect

We used Hedges' *g* to determine the magnitude of intervention effects. We calculated Cohen's *d* with the primary data (e.g., means and standard deviations, mean difference) reported for experimental and control groups in studies using Campbell web‐based effect size calculator,^6^ and then calculated Hedges' *g* with Comprehensive Meta Analysis V2.

#### 4.3.5 Unit of analysis issues

We considered the unit of analysis of the studies to determine whether individuals were randomized in groups (i.e., cluster‐randomized trials), whether individuals may have undergone multiple interventions, whether there were multiple treatment groups and whether several studies are based on the same data source.

− Clustered assignment of treatment. Cluster randomized trials included in this review were checked for consistency in the unit of allocation and the unit of analysis, as statistical analysis errors can occur when they are different.
− Multiple intervention groups and multiple interventions per individual. Studies with multiple intervention groups with different individuals (e.g., different homework modalities such as online vs. book‐based) were included in this review, although only intervention and control groups that meet the eligibility criteria will be used in the data synthesis.

#### 4.3.6 Dealing with missing data

If there were any missing data, we contacted the author at least twice to obtain additional information, if the correspondence address was available. If these data were unavailable, we only analyzed the available data; studies with missing data are described in the results section. Furthermore, the potential impact of missing data on comprehensive estimates are considered in the discussion.

#### 4.3.7 Assessment of heterogeneity

Forest plots were used to visually investigate overlaps in the confidence intervals (CIs) of the results of the individual studies. The *χ* ^2^ test was performed, and the *Q* statistics, *I* ^2^, and *τ* ^2^ index were adopted to evaluate heterogeneity across studies. For *Q* statistics, a *p*‐value of 0.05 was used as a threshold for statistical significance. The *I* ^2^ index refers to the truly observed variation ratio (Borenstein et al., 2009), and 25%, 50%, and 75% of the *I* ^2^ indicate low medium, and high heterogeneity (Higgins & Thompson, 2002). And *τ* ^2^ is the between‐studies variance (the variance of the effect size parameters across the population of studies), that is, the variance of true effect sizes (across an infinite number of studies).

#### 4.3.8 Assessment of reporting biases

Visual funnel plots with trim and fill and Egger's test of funnel plot symmetry were performed to evaluate potential publication bias.

#### 4.3.9 Data synthesis

Due to the small number of included studies, we conducted a meta‐analysis to evaluate the effectiveness of homework as compared to no‐homework. We also used a narrative approach to describe the findings, which indicated that the effect of homework depended on the time spent on it (more homework compared to less homework).

In our meta‐analysis, we encountered a variety of outcome indices. To address this, we extracted all the raw data and calculated Cohen's *d* using the Campbell web‐based effect size calculator, and then adopted Hedges's *g* as the effect size index when performing meta‐analysis. A random effects model (REM) was adopted to estimate the effect of homework compared to no‐homework. All these processes were performed using Comprehensive Meta Analysis V2.

#### 4.3.10 Subgroup analysis

According to our protocol, we planned to conduct the subgroup analysis by gender, grade level, region, publication year, mode of homework, type of homework, the measure of academic performance, and the subject (Guo et al., 2021). However, due to the limited studies included in each group, we were unable to conduct all the planned subgroup analyses.

#### 4.3.11 Sensitivity analysis

As per the protocol, we used the “one‐leave‐out” method to check the outliers that potentially influence the effect of homework compared to no‐homework.

#### 4.3.12 Treatment of qualitative research

All qualitative research was excluded.

### 4.4 Summary of findings and assessment of the certainty of the evidence

All the primary studies included in this review were rated as low quality, and as such we have refrained from assessing the overall quality of evidence.

## 5 RESULTS

### 5.1 Description of studies

#### 5.1.1 Characteristics of included studies

**Figure 2:**
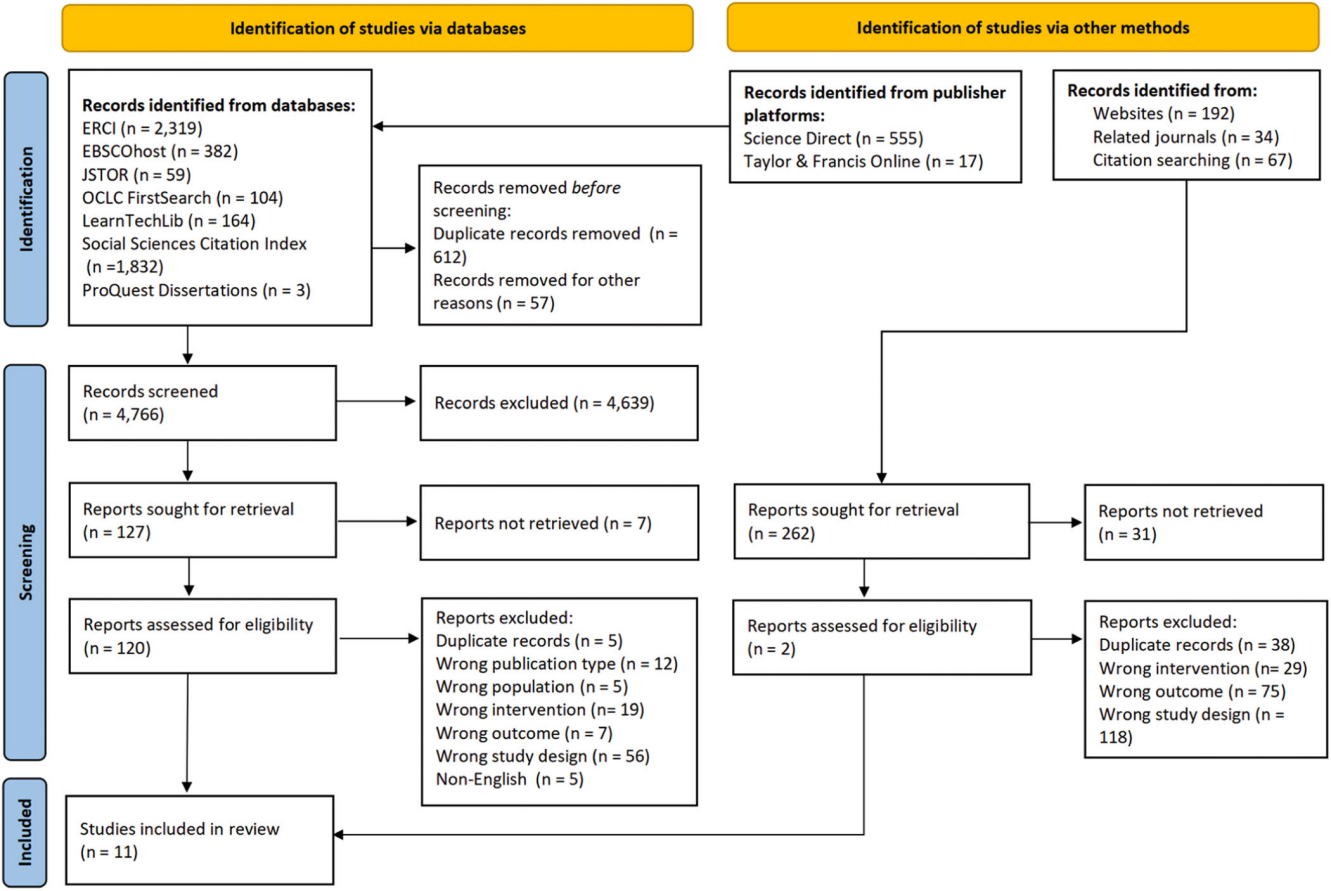
PRISMA diagram of selection process.

Of the 4904 studies identified as related to homework time and academic achievement, the titles and abstracts of 4769 were double‐screened, after removing duplicates; 127 were selected for full‐text review, and 11 primary studies were included in the systematic review (see Figure 2). The characteristics of the included studies, including study design, research setting, participants, interventions and comparisons, and outcomes, are shown in Table 2.

**Table 2:** The characteristics of included studies.

Dolean and Lervag (2021)	
Study design	Randomized controlled trial
Research setting	20 classes at six public elementary schools in Romania; Math and Writing
Participants	440 second‐grade students; 225 boys; Age: Mean = 100 months, SD = 3.97
Interventions	Description: The homework assignments were designed by a team of researchers and elementary school teachers according to the Romanian national standards. Each daily homework assignment fit on one double‐sided paper (two pages) and the requirements were identical for all students. Allocation: Students were allocated in three groups, and received different amounts of homework in writing and math. Students from the first group (Writing group) received more homework in writing and less in math, students from the second group (Balanced group) received an estimated similar amount of homework in writing and math, and students from the third group (Math group) received more homework in math and less in writing. Duration: 6 weeks Follow‐up: 4 months after the program
Outcomes	Editing; Spelling; Math fluency
Koch (1965)	
Study design	Non‐randomized controlled trial (quasi‐experimental design)
Research setting	3 classes; Arithmetic
Participants	169 sixth‐grade students
Interventions	Description: The exercises were generally of a straight computational nature, and about one‐third of the assignments consisted of “story problems” that provided practice on all the operations with whole numbers and fractions. Weekly conferences with the classroom teachers determined the content of the homework assignments. These were produced and corrected by the experimenter. Allocation: Whole duplicated page of homework given to the Full Group (received the long daily homework assignments) could be cut in half and the top half of the page given to the Half Group (which received the short daily homework assignments) as its assignment. Control Group students received no homework. Duration: 10 weeks Follow‐up: None
Outcomes	Arithmetic concept; arithmetic problem solving
Foran and Weber (1939)	
Study design	Non‐randomized controlled trial (quasi‐experimental design)
Research setting	7 classes at 7 parochial schools; Arithmetic
Participants	292 seventh‐grade students
Interventions	Description: The homework was connected with the newly introduced topics in the seventh grade, but some dealt with remedial work carried on simultaneously with the other instruction. The tasks assigned were not to exceed one‐half hour in duration but other details were left to the discretion of the teachers. All homework adhered to the conventional pattern. Allocation: All of the experimental classes had regularly assigned homework and no homework was to be assigned in any subject except arithmetic, and the tasks assigned were not to exceed one‐half hour. The control classes had no homework in any subject. The classes exchanged roles at the end of the first semester. Duration: 1 year Follow‐up: None
Outcomes	Arithmetic problems; Arithmetic computation
Foyle (1990)	
Study design	Non‐randomized controlled trial (classroom field experiment)
Research setting	4 classes at 2 public schools in the United States; Social studies
Participants	64 fifth‐grade students
Interventions	Description: Two kinds of homework were designed: practice homework and preparation homework, and the homework contained four questions per assignment. Allocation: The students were divided into three groups, and were randomly assigned to no‐homework condition, practice homework condition, and preparation homework condition. In addition to regular classroom work, the experimental groups were also given two homework assignments per week for the duration of the experimental phase, which lasted during the fall semester. Homework assignments were regularly assigned, clearly stated, regularly collected, and promptly graded and returned, with researchers recording the grades obtained by the subjects. Duration: 4 months Follow‐up: None
Outcomes	Social Studies
Gray and Allison (1971)	
Study design	Non‐randomized controlled trial (quasi‐experimental design)
Research setting	2 classes from a middle‐class suburban area school; Arithmetic
Participants	55 sixth‐grade students
Interventions	Description: Homework consisted of 20 min of practice based on material covered in class. Parents were requested to cooperate by giving no help to the children during the experiment. Students were asked to keep a record of time spent on their homework. The completed homework assignments were sent to the authors for correction and were then returned to the children. Allocation: The students were randomly assigned to two groups within each class, the experiment extended through a period of 8 weeks. One group student was assigned three 20‐min homework per week, the other group students received regular classroom instruction with no homework assignments. At the end of the first 4 weeks, the treatments were reversed so that all children received both treatments within each class. Duration: 8 weeks Follow‐up: None
Outcomes	Arithmetic computation, arithmetic reasoning, and arithmetic understanding
Maertens (1969)	
Study design	Randomized controlled trial
Research setting	12 classes at 4 public schools from Osseo in Minnesota; Arithmetic
Participants	319 third‐grade students
Interventions	Description: Two approaches of homework were used in the intervention groups. One is teacher‐prepared homework which assigned by teacher in accordance with their normal classroom procedures. There was no standards or limitations to guide the teacher, and the amount and type of homework varied as the teacher perceived needs. Another is experimenter‐prepared homework which required the regular assignment of experimenter‐prepared arithmetic homework in conjunction with the completion of certain. These daily assignments were designed to provide drill upon materials that had been covered in class, facility with verbal problems, and experience with problem situations requiring the use of the generalizations stressed in the unit under study. Allocation: Pupils were randomly assigned to classrooms within each of the schools. The students in comparison classes received no homework in arithmetic, and no arithmetic materials were allowed to be taken home, nor was a child permitted to finish daily assignments at home. Duration: 7 months Follow‐up: None
Outcomes	Knowledge of arithmetic processes, Arithmetic computational skill, and Arithmetic problem‐solving ability
Maertens and Johnston (1972)	
Study design	Non‐randomized controlled trial (quasi‐experimental design)
Research setting	School district of Sweet Home from Oregon in Minnesota; Arithmetic
Participants	417 students, including146 fourth‐grade students, 137 fifth‐grade students and 134 sixth‐grade students
Interventions	Description: Two treatments of homework were used in the intervention groups. One is per problem knowledge of results, which assigned daily homework in arithmetic to children on Monday, Tuesday, Wednesday and Thursday. These homework materials, prepared by the experimenters were carefully designed to complement and enrich the materials covered in the students' textbooks. Each arithmetic assignment provided drill upon materials previously covered in class, and provided children with the opportunity of applying the mathematical principles which had been learned in verbal problem situations. Another is delayed knowledge of results, which assigned homework identical to those in the per problem treatment and followed identical procedures, except that children did not receive knowledge of results until the entire homework assignment was completed. At that time, the parent read the correct answers to each of the questions. Allocation: Children were randomly assigned to treatments. The students in comparison group were not given any arithmetic homework. Further, they were not allowed to complete unfinished daily assignments at home, nor were they given any additional homework assignments from other subject areas to compensate for their lack of arithmetic homework. Duration: 6 weeks Follow‐up: None
Outcomes	Arithmetic computational skill and arithmetic problem‐solving ability
Townsend (1995)	
Study design	Non‐randomized controlled trial (quasi‐experimental design)
Research setting	2 classes; Vocabulary lessons
Participants	40 third‐grade students; Age: 8–9 years old
Interventions	Description: Homework designed to students, including finding definitions in the dictionary, illustrating vocabulary words, writing paragraphs using the words, answering yes or no questions based on the understanding of the words, and synonym worksheets. Allocation: 40 students from two classes were involved in the experiment. One class was given homework each night to reinforce the new vocabulary learned that day. The other sample of students were not assigned any reinforcing homework. At the end of 3 weeks, a teacher‐made test was administered to assess the acquisition of the new vocabulary words. Duration: 3 weeks Follow‐up: None
Outcomes	vocabulary knowledge and understanding
Ewart Anderson (1946)	
Study design	Non‐randomized controlled trial (quasi‐experimental design)
Research setting	One class; English, Social studies, and Mathematics
Participants	58 eighth‐grade students
Interventions	Description: Unclear Allocation: 58 students were placed into experimental and control group study based on the scores of Otis Self‐Administering Test of Mental Ability. Experimental group students were arranged the home study work supplied with assignment sheets covering the amount of work to be studied, that which was to be reported on specifically, and directions for study. The control group students received no homework. Both groups of pupils had the same teachers in three subjects used. Duration: Unclear Follow‐up: None
Outcomes	Unite test of English, Social studies, and Mathematics subjects
McGrath (1992)	
Study design	Non‐randomized controlled trial (quasi‐experimental design)
Research setting	3 classes in University High School in San Diego; Senior English
Participants	54 students, including 25 male and 29 female
Interventions	Description: A letter was sent to the students' parents with a self‐addressed stamped postcard requesting the parents' consent for their sons or daughters to participate in a 15‐day research experiment in Senior English. The parents of students in three senior English classes (Survey of English Literature) consented to allow their sons and daughters to participate in either a control group that received no homework or an experimental group that received homework. Allocation: The sample of students was divided into the two groups of 47 students each by the alphabetic listing of his/her last name. Duration: 15 days Follow‐up: None
Outcomes	English literature
Tupesis (1972)	
Study design	Non‐randomized controlled trial (quasi‐experimental design)
Research setting	2 classes in Monona Grove High School; Senior English
Participants	43 students, including 15 male and 28 female
Interventions	Description: The manipulated variable is out‐of‐class homework – its presence (T2) or absence (T1), and the order in which the remaining instructional tasks within each treatment are sequenced. The daily lecture, discussions stemming from the lecture, examples, and the oral exercises did not vary across the two treatments. Allocation: The subjects have been “computer randomized” from all those students selecting to take Geometry B at Monona Grove during the 1971–1972 academic year. Duration: 9 weeks Follow‐up: None
Outcomes	Mathematics subjects

##### Study designs

Two included studies used a randomized controlled trial (Dolean & Lervag, 2021; Maertens & Johnston, 1972). The other nine studies adopted quasi‐experimental designs (Ewart Anderson, 1946; Foran & Weber, 1939; Foyle, 1990; Gray & Allison, 1971; Koch, 1965; Maertens & Johnston, 1972; McGrath, 1992; Townsend, 1995; Tupesis, 1972) (Foran & Weber, 1939; Foyle, 1990; Koch, 1965). All of these nine studies adopted the class as the unit by which to assign individual participants to treatment or control arms. Only one of the eleven total studies included follow‐up data (Dolean & Lervag, 2021).

##### Research setting

One of the nine studies was published in 2021 (Dolean & Lervag, 2021); the remaining studies were conducted between 1939 and 1995 (Ewart Anderson, 1946; Foran & Weber, 1939; Foyle, 1990; Gray & Allison, 1971; Koch, 1965; Maertens & Johnston, 1972; Maertens, 1969; Townsend, 1995). The experiments in three of the nine studies were conducted in between two and six public primary schools (Dolean & Lervag, 2021; Foyle, 1990; Maertens, 1969). The treatment groups in the study by Foran and Weber (1939) were set in seven parochial schools. These are private schools affiliated with a religious entity (Foran & Weber, 1939). The participants in Gray and Allison (1971) and Tupesis (1972) were recruited from a middle‐class suburban area school (Gray & Allison, 1971; Tupesis, 1972), and in McGrath (1992) participants were from University High School (McGrath, 1992). The research setting in the remaining studies was not reported clearly (Ewart Anderson, 1946; Koch, 1965; Maertens & Johnston, 1972; Townsend, 1995).

##### Participants

The unique number of participants ranged from 40 to 417. Four of the studies included over 100 participants (Dolean & Lervag, 2021; Foran & Weber, 1939; Maertens & Johnston, 1972; Maertens, 1969). The average sample size of the remaining studies was 53.4.

Eight papers reported the grade of the subjects: one study focused on 2nd grade students (Dolean & Lervag, 2021), two on 3rd grade (Maertens, 1969; Townsend, 1995), one on 5th (Foyle, 1990), two on 6th (Gray & Allison, 1971; Koch, 1965), one on 8th students (Ewart Anderson, 1946), and one focused on all three grades from 4th to 6th (Maertens & Johnston, 1972). Nine studies reported the number of classes, which ranged from 2 classes (Ewart Anderson, 1946; Gray & Allison, 1971; Townsend, 1995; Tupesis, 1972) to 12 (Maertens, 1969).

Four experiments also considered participants' intelligence. In Koch (1965), the mean IQ scores for experimental and control groups were similar in both language (control group: 111, half group: 109, and full group: 112) and non‐language groups (control group: 109, half group: 113, and full group: 109) (Koch, 1965). Intelligence was excluded from the potential confounding factors in Foran and Weber (1939), who reported the mean of intelligence test scores in two groups as 105.55 (SD = 9.20) and 107.71 (SD = 9.17), respectively (Foran & Weber, 1939). Maertens (1969) included the level of intelligence as a classification variable in the data analysis. Students were divided into three groups based on their IQ: high (IQ of 112 and above), middle (IQ of 103 through 111), and low group (IQ of 102 and below). The median IQ of the total sample was 106 (Maertens, 1969). Tupesis (1972) tested the IQ in two groups using Henmon‐Ne1s and found no difference between them (108.5 vs. 109.1) (Tupesis, 1972).

##### Interventions and comparisons

All 11 interventions were pencil‐paper homework designed by teachers or experimenters, but the content varied depending on the target subject. None of the studies regarded homework duration as the primary variable, and only two studies described the exact duration in the experimental groups. Gray and Allison (1971) clearly defined that the students in the intervention group received three 20‐min homework assignments per week, and the students in the control group received no homework (Gray & Allison, 1971). Dolean and Lervag (2021) reported the homework time in three groups (writing group, balanced group, and math group) as an average of 27, 20, and 21 min, respectively (Dolean & Lervag, 2021). Foran and Weber (1939) limited homework time to within half an hour, when teachers designed the regular homework for experimental classes.

Dolean and Lervag (2021) allocated students to three groups with different amounts of homework in writing and math: a writing group (students received more homework in writing and less in math), balanced group (students received similar amounts of homework in writing and math), and math group (students received more homework in math and less in writing); the balanced group was a potential comparison (Dolean & Lervag, 2021). The remaining studies simply assigned homework to experimental groups and used the students with no homework as their comparisons.

Two studies distinguished the different types of homework. Foyle (1990) designated practice homework and preparation homework, which were randomly assigned to two of three treatment groups (Foyle, 1990). Maertens and Johnston (1972) considered the time of feedback and then proposed two homework treatments. One was homework with immediate feedback: children were assigned daily homework and received the answer to each problem or exercise as it was completed. The other was homework with delayed feedback: children were assigned homework identical to that in the immediate feedback treatment but did not received the answers until after the entire homework assignment was completed (Maertens & Johnston, 1972).

Ten of 11 publications reported the duration of the interventions, ranging from 3 weeks to 1 year (Dolean & Lervag, 2021; Foran & Weber, 1939; Foyle, 1990; Gray & Allison, 1971; Koch, 1965; Maertens & Johnston, 1972; Maertens, 1969; McGrath, 1992; Townsend, 1995; Tupesis, 1972). Only one study conducted a follow‐up survey, 4 months after the program (Dolean & Lervag, 2021).

##### Outcomes

The outcomes varied depending on the research subjects. Five of the primary studies focused on the effectiveness of homework on arithmetic skills, especially on arithmetic concepts (Gray & Allison, 1971; Koch, 1965), computations (Foran & Weber, 1939; Gray & Allison, 1971; Maertens & Johnston, 1972; Maertens, 1969) and problem solving (Foran & Weber, 1939; Koch, 1965; Maertens & Johnston, 1972; Maertens, 1969). Dolean and Lervag (2021) also mentioned the effect on math; they only included math fluency results. They further considered the effect on writing and used editing and spelling performance as their outcomes (Dolean & Lervag, 2021).

Townsend (1995) researched the effect of homework on vocabulary lessons, using vocabulary knowledge and understanding as outcomes (Townsend, 1995). Foyle (1990) studied the effectiveness of practicing homework on performance in the social studies lessons (Foyle, 1990). Ewart Anderson (1946) also explored the average effect of homework on English, social studies, and math, and adopted a unified test score as the final outcome (Ewart Anderson, 1946). McGrath (1992) estimated the effectiveness of homework on English literature (McGrath, 1992).

#### 5.1.2 Excluded studies

Studies were excluded for the following reasons: (1) they did not include a homework intervention, (2) they did not assess academic achievement, (3) they did not include K‐12 students, or (4) did not use an experimental design. We scanned 42 primary studies included in the EEF toolkit on the homework and 40 of them were excluded. The details are shown in Table 3.

**Table 3:** Excluded studies.

ID	Title	Reason
Al‐Naqbi (2014)	The effects of instructional homework technique on chemistry achievement of the United Arab Emirates male and female tenth graders	No homework time
Bailey (2006)	Interactive homework: A tool for fostering parent–child interactions and improving learning outcomes for at‐risk young children	No homework time
	The differential effect of basic mathematics skills homework via a web‐based intelligent tutoring system across achievement subgroups and mathematics domains: A randomized field experiment	No homework time
Bell (2015)	An investigation of the impact of a flipped classroom instructional approach on high school students' content knowledge and attitudes toward the learning environment	Wrong intervention
Chao et al. (2015)	Exploring students' learning attitude and achievement in flipped learning supported computer aided design curriculum: A study in high school engineering education	Wrong intervention
Clark (2013)	Examining the effects of the flipped model of instruction on student engagement and performance in the secondary mathematics classroom: An action research study	Wrong intervention
Dadas (1976)	A study of the effects of assigning spiral exploratory homework upon achievement in and attitude towards mathematics	No homework time
Davis (2004)	The impact of parental involvement: a study of the relationship between homework and kindergarten Texas Primary Reading Inventory scores	Wrong population
Duffy (2016)	The impact of flipped learning on student achievement in an eight grade earth science classroom	Wrong intervention
Esperanza et al. (2016)	Flipped classroom model: Effects on performance, attitudes and perceptions in high school algebra	Wrong intervention
Flansburg (2016)	Flipped learning instruction: Differentiating mathematics instruction through the use of technology	Wrong intervention
Flick (2019)	The effects of flipped learning in the sixth‐grade mathematics classroom	Wrong intervention
Foyle (1984)	The effects of preparation and practice homework on student achievement in tenth‐grade American history	Overlapped with Foyle (1990)
Freet (2016)	Flipping the classroom: An exploration of the effect of inverted learning on student achievement in a high school mathematics classroom	Wrong intervention
Glynn (2013)	The effects of a flipped classroom on achievement and student attitudes in secondary chemistry.	Wrong intervention
Howell (2013)	Effects of an inverted instructional delivery model on achievement of ninth‐grade physical science honors students	Wrong intervention
Hungi and Ngware (2017)	Investigating the effects of community‐based interventions on mathematics achievement of girls from low‐income households in Kenya	Wrong intervention
Kiesner (1997)	The effects of a parental homework monitoring intervention on school engagement of high risk middle school students	No homework time
Kırmızı and Kömeç (2019)	The impact of the flipped classroom on receptive and productive vocabulary learning	Wrong intervention
Kirvan et al. (2015)	Flipping an algebra classroom: Analyzing, modeling, and solving systems of linear equations	Wrong intervention
Lai et al. (2020)	The effectiveness of team‐based flipped learning on a vocational high school economics classroom	Wrong intervention
Lonigan and Whitehurst (1998)	Relative efficacy of parent and teacher involvement in a shared‐reading intervention for preschool children from low‐income backgrounds	Wrong population
Meloy (1987)	Effects of homework on language arts achievement in third and fourth‐grades	Missing data
(Metcalf (2015)	The impact of flipping a middle school classroom on student achievement	Wrong intervention
Montgomery (2015)	The effects of flipped learning on middle school students' achievement with common core mathematics	Wrong intervention
Nordstrom (2012)	The impact of online and traditional homework on the attitudes, achievement, and learning styles of sixth grade language arts students	No homework time
Özcan and Erktin (2015)	Enhancing mathematics achievement of elementary school students through homework assignments enriched with metacognitive questions	No homework time
Ramaglia (2015)	The flipped mathematics classroom: a mixed methods study examining achievement, active learning, and perception	Wrong intervention
Ripley (2015)	An examination of flipped instructional method on sixth graders' mathematics learning: Utilizing propensity score matching.	Wrong intervention
Robledo‐Ramón and García‐Sánchez (2013)	Strategy instruction for writing composition at school and at home	Wrong intervention
Saunders (2014)	The flipped classroom: Its effect on student academic achievement and critical thinking skills in high school mathematics	Wrong intervention
Schultz et al. (2014)	Effects of the flipped classroom model on student performance for advanced placement high school chemistry students	Wrong intervention
Schwankl (2013)	Flipped classroom: Effects on achievement and student perception	Wrong intervention
Smith (2015)	The efficacy of a flipped learning classroom	Wrong intervention
Špilka (2014)	Pedagogical experiment with online visualization of mathematical models in math teaching on elementary school	Wrong intervention
Tamayo (1992)	Hispanic parent monitoring of seventh‐grade mathematics homework assignments and relationship with achievement and self‐esteem	No homework time
Tsai et al. (2015)	The effects of problem‐based learning with flipped classroom on elementary students' computing skills: A case study of the production of ebooks	Wrong intervention
Van Voorhis (2010)	Adding families to the homework equation: A longitudinal study of mathematics achievement	No homework time
Wiginton (2013)	Flipped instruction: An investigation into the effect of learning environment on student self‐efficacy, learning style, and academic achievement in an algebra 1 classroom	Wrong intervention
Yousefzadeh (2015)	The effect of flipped learning (revised learning) on Iranian students' learning outcomes	Wrong intervention

### 5.2 Risk of bias in included studies

#### 5.2.1 Risk of bias of RCTs

**Figure 3:**
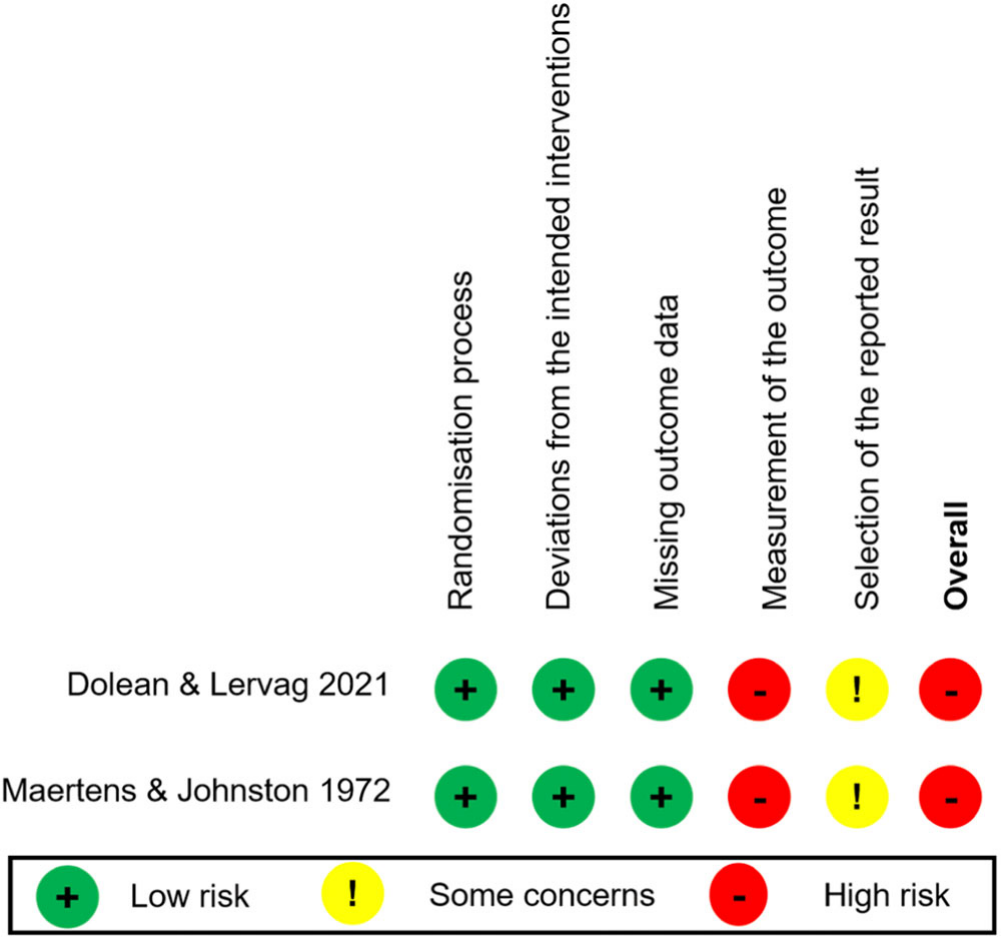
Risk of bias of RCTs included in the review.

Two of 11 studies used randomized controlled trials, both of which were evaluated as having high risk of bias based on the ROB2 (Dolean & Lervag, 2021; Maertens & Johnston, 1972), especially in terms of bias in the measurement of the outcome (details are shown in Figure 3).

#### 5.2.2 Risk of bias of NRCTs

**Figure 4:**
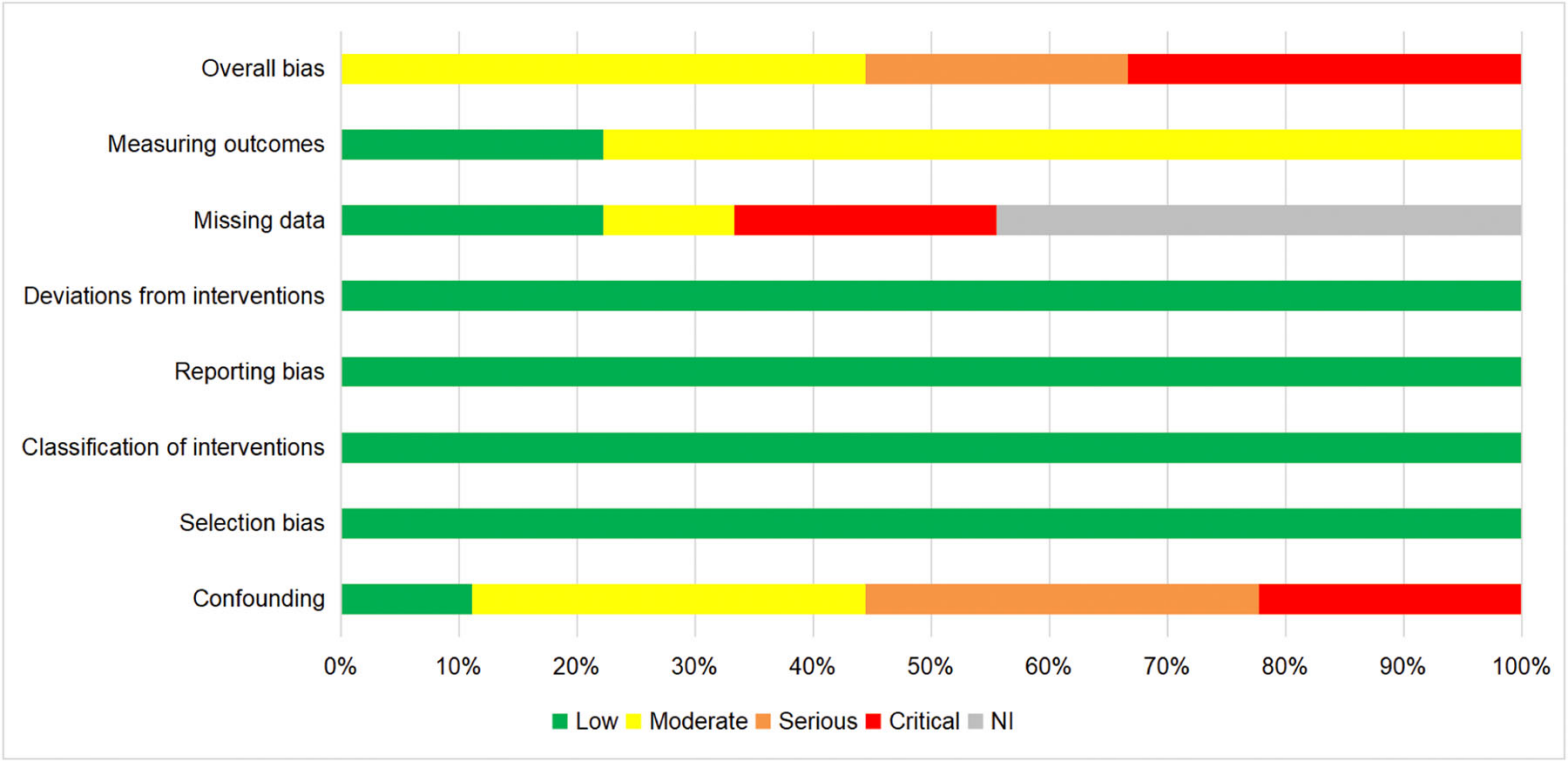
Risk of bias of non‐RCTs included in the review.

**Figure 5:**
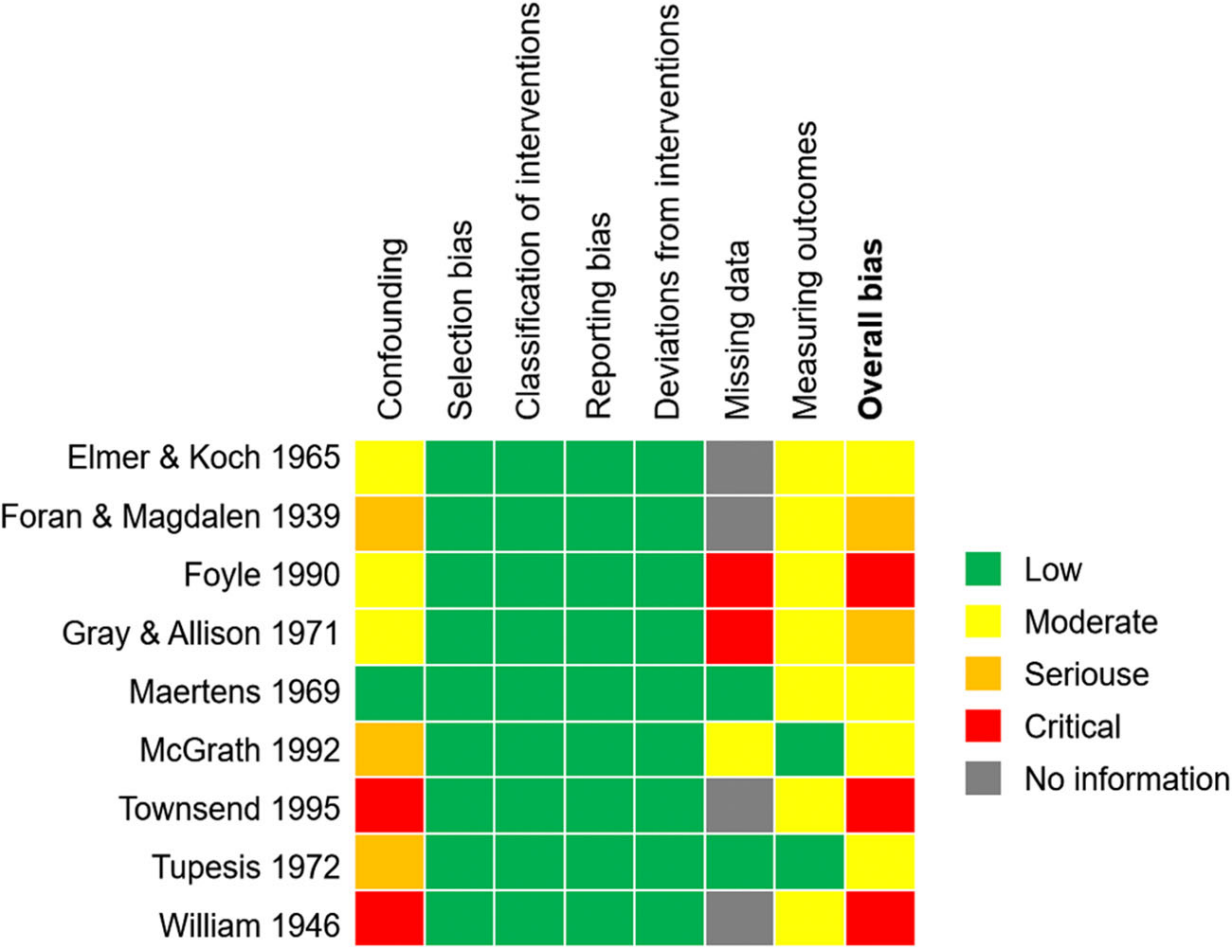
The summary of risk of bias of non‐RCTs.

Nine of 11 studies adopted non‐randomized controlled designs, none of which were assessed as of low risk of bias; 4 had moderate risk of bias (Koch, 1965; Maertens, 1969; McGrath, 1992; Tupesis, 1972), 2 in serious risk of bias (Foran & Weber, 1939; Gray & Allison, 1971), and 3 had critical risk of bias (Ewart Anderson, 1946; Foyle, 1990; Townsend, 1995). Specifically, as shown in Figure 4, all of these studies were at low risk of bias in four domains (selection bias, classification of interventions, reporting bias and deviations from interventions), moderate risk of bias in measuring outcomes, and critical risk of confounding and missing data (details are shown in Figure 5).

### 5.3 Effects of interventions

Eleven publications were included in the present review, and the outcomes were divided into two groups based on the comparison: one comparison is homework versus no homework and another is more homework versus less homework. Fewer studies were included in the second comparison and could not be synthesized quantitatively. Thus, we provide a meta‐analysis of the first comparison outcome and a narrative description of the second.

#### 5.3.1 Homework versus no‐homework

##### Overall effect of homework

Ten studies with 14 independent reports compared the effectiveness of homework with no‐homework treatment. Seven of them revealed an advantage of homework groups in academic performance (Ewart Anderson, 1946; Foran & Weber, 1939; Foyle, 1990; Gray & Allison, 1971; Maertens & Johnston, 1972; Townsend, 1995; Tupesis, 1972). The other three suggested no difference was present between experimental and control groups (Koch, 1965; Maertens, 1969; McGrath, 1992).

**Figure 6:**
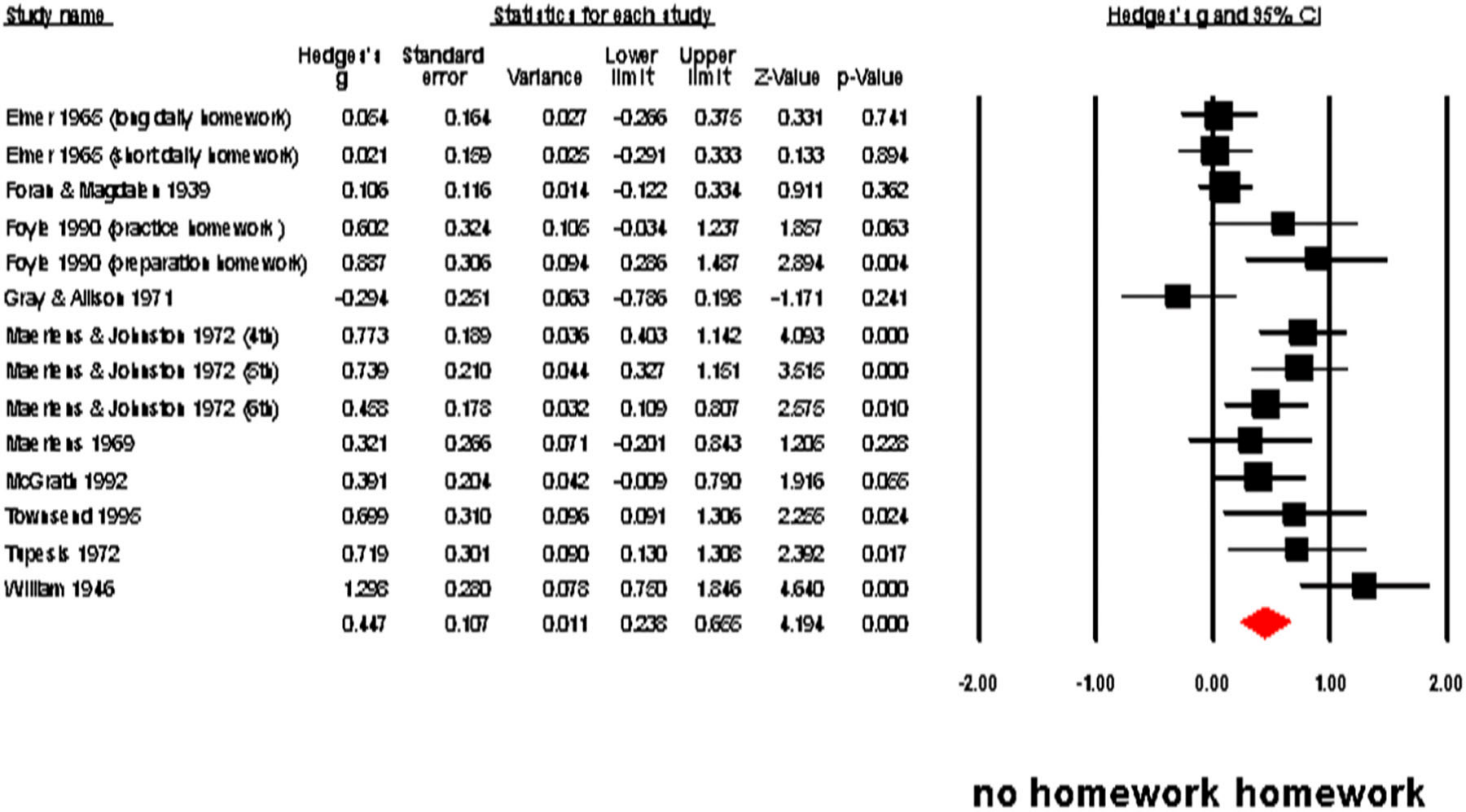
The effectiveness of homework on academic achievement.

We analyzed the comprehensive effect of homework using a random‐effect meta‐analysis, as shown in Figure 6, homework had a significant positive effect on students' academic achievement (*n* = 14; *g* = 0.45, 95% CI: 0.24–0.66; *Q* = 454.30, *I* ^2^ = 71.30%, *τ* ^2^ = 0.11), especially in arithmetic computation (*n* = 5; *g* = 0.46, 95% CI: 0.17–0.75; *Q* = 13.03, *I* ^2^ = 69.29%, *τ* ^2^ = 0.07) and arithmetic problems solving (*n* = 6; *g* = 0.17, 95% CI: 0.02–0.33; *Q* = 6.87, *I* ^2^ = 27.17%, *τ* ^2^ = 0.01), but not in arithmetic concepts (*n* = 3, *g* = −0.02, 95% CI: −0.22–0.18; *Q* = 1.46, *I* ^2^ = 0.00%, *τ* ^2^ = 0.00).

##### Publication bias

**Figure 7:**
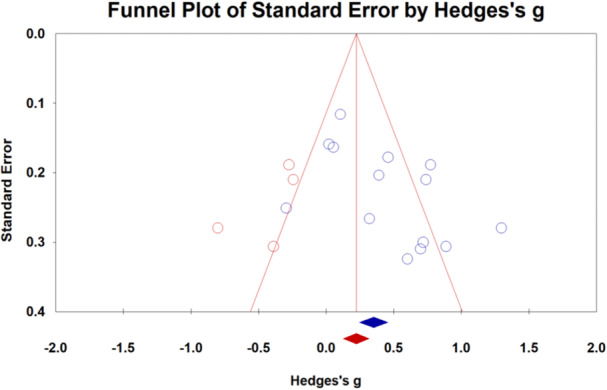
Funnel plot of the effectiveness of homework.

As shown in Figure 7, all effect size was symmetrically distributed, and the results of the meta‐analysis had no obvious change before and after trim and fill. The result of Egger's test also showed no significant publication bias (*t* = 2.20, *p* = 0.06).

##### Sensitivity test

**Figure 8:**
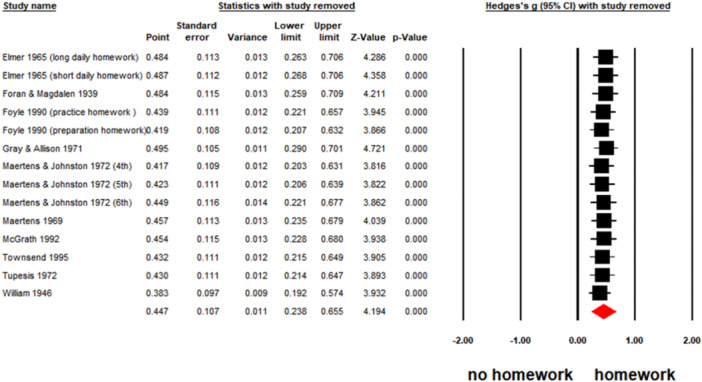
The forest plot for sensitivity test.

We performed a sensitivity test using the “one‐leave‐out” method, and found no outliers. The effect sizes were from 0.383 to 4.484 (Figure 8).

#### 5.3.2 More homework versus less homework

Two publications explored the effectiveness of homework according to its duration (Dolean & Lervag, 2021; Koch, 1965). As mentioned above, Koch (1965) compared homework with no homework and the difference between long daily homework and short daily homework in their effect on developing arithmetic concepts and problem‐solving. The results indicated that students who were assigned longer homework tasks (approximately 30 min) achieved greater growth in their understanding of arithmetic concepts compared to those who had half the amount of homework (about 15 min). The mean difference in test scores was 8.577 versus 3.448 (*F* = 11.944, *p* < 0.001). However, no such difference was observed in arithmetic problem‐solving (Koch, 1965).

Although we observed the positive impact of homework duration on performance in arithmetic concepts from this experiment, the assignments for both the long homework group and the half homework group were relatively short compared to the recommended daily averages of 40 min for primary school students in the United States, 90–120 min for Primary 5 and 6 students in Singapore, and a maximum of 60 min for Grades 3 to 6 in China. This discrepancy might indicate a floor effect of homework time. Based solely on this experiment, we could not establish an optimal homework time. Further research, possibly incorporating a broader range of homework duration and a larger sample size, would be necessary to establish more precise guidelines.

In 2021, Dolean and Lervag tested the impact of variations of homework amount assigned in elementary school on the academic achievement of students. They divided students into three groups that received different amounts of homework in writing and math for 20 days: a writing group, a balanced group (with both writing and math homework), and a math group. The average time to complete the homework was significantly higher for the students from the writing group (mean 27 min: SD = 9.41, range: 9–84) as compared to the students from the balanced group (20 min: SD = 7.09, range: 8–53) and the Math group (21 min: SD = 8.75, range: 8–77). The results revealed a significant direct effect of homework quantity on writing ability. Compared with the writing group, the short‐term effect of less homework on editing and spelling was worse in the Math group (*d* = −0.31 and –0.346), but the effect was no longer clear after 4 months. However, increasing or decreasing the amount of math homework did not alter its impact on math skill (Dolean & Lervag, 2021).

Overall, the experimental findings provide partial evidence that appropriate homework contributes positively to academic achievement among K‐12 students. However, the optimal time allocation for homework is still unclear. Unfortunately, the planned subgroup analyses were not feasible due to insufficient data. Consequently, potential differences across genders, grades, subjects, and regions remain untested, and the moderating and mediating variables remain unidentified.

## 6 DISCUSSION

### 6.1 Summary of main results

The present review identified 11 publications that used experimental methods to assess the relationship between homework duration and academic outcomes. We grouped the studies into two categories: homework versus no homework, and long homework versus less homework. Given the limited number of studies and the variability in outcomes, we employed two approaches to present the results: a meta‐analysis for comparison between assigning homework and not assigning homework, and a narrative synthesis for the comparison between assigning longer homework and less homework.

Ten articles compared students' academic performance in a homework group with that in a no‐homework group (Ewart Anderson, 1946; Foran & Weber, 1939; Foyle, 1990; Gray & Allison, 1971; Koch, 1965; Maertens & Johnston, 1972; Maertens, 1969; McGrath, 1992; Townsend, 1995; Tupesis, 1972). Compared with no homework, students who received homework obtained higher test scores (*g* = 0.45), particularly in arithmetic computation (*g* = 0.46) and problem‐solving (*g* = 0.17).

Two experiments explored the effectiveness of homework moderated by the homework time (Dolean & Lervag, 2021; Koch, 1965). In Koch (1965), the effect of long‐daily homework (20 to 30 min) and short‐daily homework (10–15 min) were compared. He suggested that achievement in arithmetic concepts following daily long homework assignments was higher than that with short homework assignments. Recently, Dolean and Lervag (2021) confirmed the effect of homework on writing skill. Their results also support Koch (1965), in that increased time spent on homework (average 20 min) fostered higher gains in writing achievement (Dolean & Lervag, 2021).

We planned to conduct a subgroup analysis to examine the differences in homework effectiveness across factors such as gender, grade level, subject matter, and geographical regions. However, due to the scarcity of studies available for each subgroup, we could not execute all the planned subgroup analyses.

Additionally, we aimed to identify potential factors on the duration of homework, including the academic subject, task difficulty, type of homework, mode of homework, parental involvement, and feedback on homework. However, only two studies investigated the factors that could affect the effectiveness of homework. Foyle (1990) explored the difference between types of homework and found no significant disparities between groups that received either preparation‐ or practice‐oriented homework (Foyle, 1990). Similarly, Maertens and Johnston (1972) examined the moderated effect of feedback and observed no obvious distinction in the effects of homework between students who received immediate feedback (per problem) and those who received delayed feedback (delayed until after the homework was fully complete) (Maertens & Johnston, 1972).

### 6.2 Overall completeness and applicability of evidence

While we developed the search strategy under the guidance of information specialists, certain words, such as “attainment,” were not included in the keywords for academic performance. This might have resulted in missing some relevant studies. Our search did not impose any language restrictions, however we did not include non‐English databases, such as the widely‐used Chinese database, CNKI. This could potentially limit the applicability of our conclusion in non‐English contexts. Our most recent search was conducted in November 2021, more than 2 years before the publication of this review. This time gap has affected the current relevance of our evidence.

Only one study was published online recently in 2021, while the remaining studies spanned from 1939 to 1995. This may reflect the changing attitudes towards homework over time. In the early 20th century, educators widely believed that homework contributed to creating disciplined minds. However, by 1940, concern arose that homework might interfere with other home activities, leading to a backlash against its practice. This trend shifted in the late 1950s when the U.S. government was concerned that their education lacked rigor. Schools then viewed more rigorous homework as a partial solution to this problem. However, in the 1980s, some researchers argued that excessive homework could be detrimental to students' mental health, and deprive students of essential free time (Costley, 2013; Güven & Akçay, 2019). The results of TIMSS indicates that the trend of assigning homework from 2003 to 2015 has been negative in both 4th and 8th grades in all selected countries, except in Turkey (Güven & Akçay, 2019). The attitudes toward homework have fluctuated significantly over the years, reflecting changing educational philosophies and societal concerns, which also affected the education research direction.

Overall, given the limited number of studies included in our review and the fact that many of them are outdated, our findings should be interpreted with caution.

### 6.3 Quality of the evidence

#### 6.3.1 Quality of RCTs included in the review

The two RCTs were assessed as at high risk of bias (Dolean & Lervag, 2021; Maertens & Johnston, 1972). In each domain, both studies were at low risk of bias in randomization processes, deviations from intended interventions, and missing outcome data. However, they were assessed as having a high risk in measurement of outcomes, with some risk of bias in the selection of the reported results.

As mentioned above, the method used to measure academic performance could potentially affect its relationship with homework time (Cooper et al., 2006; Fan et al., 2017). These two RCTs employed teacher‐designed tests instead of general standardized tests. Although teacher‐designed tests can be both valid and reliable assessment tools if the teachers are properly trained in test construction and understand the principles of validity and reliability, neither of the two studies reported on the validity and reliability of the questionnaires (Kinyua & Odiemo, 2018; Setiabudi et al., 2019).

#### 6.3.2 Quality of NRCTs included in the review

None of nine NRCTs were at low risk of bias, four were at moderate risk (Koch, 1965; Maertens, 1969; McGrath, 1992; Tupesis, 1972), two at serious risk (Foran & Weber, 1939; Gray & Allison, 1971), and three at critical risk (Ewart Anderson, 1946; Foyle, 1990; Townsend, 1995). All of these studies were at low risk of selection bias, classification of interventions bias, reporting bias, and bias of deviations from interventions. However, eight of nine studies were assessed having a risk of confounding bias, including three studies were at moderate risk (Foyle, 1990; Gray & Allison, 1971; Koch, 1965), three studies was at serious risk (Foran & Weber, 1939; McGrath, 1992; Tupesis, 1972), and two studies were at critical risk (Ewart Anderson, 1946; Townsend, 1995).

In contrast to RCTs, the absence of random participant assignment in quasi‐experiments can lead to a higher susceptibility to bias. This can result in the blending of intervention effects with confounding variables. Despite this, statistical techniques can be employed to control or balance these biases (Waddington et al., 2017). Out of the nine studies included in this review that used a quasi‐experimental design, only four factored in the Intelligence Quotient (IQ) before the intervention (Foran & Weber, 1939; Gray & Allison, 1971; Koch, 1965; Tupesis, 1972). Additionally, one study considered both the mental ability of students and the effectiveness of teachers (Ewart Anderson, 1946). It is important to note, however, that the impact of homework on academic achievement is influenced by a variety of confounding factors. These include the student's study skills, teacher effectiveness, socioeconomic status, parental involvement, and homework management strategies (Deysolong, 2023; Trautwein, 2003; Van Voorhis, 2003; Xu, 2009).

### 6.4 Potential biases in the review process

There were several limitations with respect to the review process. Although many studies evaluated the effectiveness of homework, few studies considered time spent on the homework. Even if studies mentioned homework duration, the time scales they covered were narrow, ranging from 10 to 30 min, which may lead to a floor effect. In addition, we tried to include all relevant studies in the selection process, but several studies were unavailable due to our limited authority over databases and lack of reply from the authors.

### 6.5 Agreements and disagreements with other studies or reviews

The main findings of this present review is that homework is beneficial for primary school students, especially in arithmetic computation and arithmetic problems‐solving skills. This conclusion is generally consistent with previous reviews that indicated a positive correlation between homework duration and academic performance (Baş et al., 2017; Cooper et al., 2006; Cooper, 1989; Fan et al., 2017). However, it is important to note that due to insufficient data, this review only included students from 2nd to 8th students. Therefore, it remains unclear whether this conclusion can be generalized to other age groups.

Considering the workload of homework, some researchers have proposed a curvilinear relationship between homework duration and academic achievement (Fernández‐Alonso et al., 2015; Keith, 1982; Krejtz et al., 2018; Reteig et al., 2019), For example, Fernández‐Alonso et al. (2015), who surveyed 7725 Spanish adolescents with an average age of 13.78 years, concluded that the optimum duration of homework was 1 h per day for mathematics and science (Fernández‐Alonso et al., 2015). However, our review included a limited number of studies with narrow homework time scales (10–30 min), we were unable to ascertain the pattern (be it linear or curvilinear) of the relationship between homework time and academic performance, much less the optimal amount of homework time.

Previous studies have also demonstrated that the relationship between homework and academic performance is influenced by factors such as the gender (Cadime et al., 2017), grade level (Baş et al., 2017), region (Zhu, 2015), publication year (Gill & Schlossman, 2004; Twenge et al., 2004), measurement tool (Fan et al., 2017), type of homework (Qiao & Fan, 2020), and subject (Fan et al., 2017; Trautwein & Lüdtke, 2007). We intended to identify the potential moderators using subgroup analysis in this review, but we were unable to do so due to insufficient data.

## 7 AUTHORS' CONCLUSIONS

Homework is recommended as a supplement to improve the academic performance of primary school students, especially in arithmetic computation and arithmetic problems‐solving skills. However, it is still uncertain how much time they should allocate each day for homework to achieve the best results. Therefore, it is essential to conduct further high‐quality experiments to explore the optimal homework duration. Furthermore, additional research is required to understand the impact of homework on preschool and secondary school students.

## CONTRIBUTIONS OF AUTHORS

- Content: Guo LP, Hu XL, Liu CY, Xing X
- Systematic review methods: Guo LP, Li XX, White H, Yang KH
- Statistical analysis: Guo LP, Hu XL, Liu CY, Xu Z
- Information retrieval: Guo LP, Li JY, Li XX, Yang KH

## DECLARATIONS OF INTEREST

All authors declared no potential interest.

## INTERNAL SOURCES

This review is supported by funding of the Major Project of the National Social Science Fund of China: Research on the Theoretical System, International Experience, and Chinese Path of Evidence‐based Social Science (No. 19ZDA142).

## DIFFERENCES BETWEEN PROTOCOL AND REVIEW

Compared to the protocol, we did an extra retrieval on the LearnTechLib (http://learntechlib.org/);

We planned to conduct the subgroup analysis by gender, grade level, region, publication year, mode of homework, type of homework, the measure of academic performance, and the subject, but due to the limited studies included in this review, we did not execute all the subgroup analyses which were listed in our protocol.

We planned to use the GRADE framework to rate the overall quality of evidence in the protocol. However, given that all the primary studies included in this review exhibited low quality according to ROB2/ROBINS‐I, it's prudent to acknowledge this limitation. Therefore, we have refrained from assessing the overall quality of evidence.

## Footnotes

1
https://classroom.google.com/.

2
https://www.fireflylearning.com/.

3
https://www.eschools.co.uk/.

4
https://moodle.org/index.php.

5 We searched this source, but it contains archival information.

6
https://www.campbellcollaboration.org/research-resources/effect-size-calculator.html

## References

[cl21431-bib-0001] Dolean, D. D. , & Lervag, A. (2021). Variations of homework amount assigned in elementary school can impact academic achievement. The Journal of Experimental Education, 90(2), 280–296. 10.1080/00220973.2020.1861422

[cl21431-bib-0002] Ewart Anderson, W. (1946). An attempt through the use of experimental techniques to determine the effect of home assignments upon scholastic success. The Journal of Educational Research, 40(2), 141–143. 10.1080/00220671.1946.10881499

[cl21431-bib-0003] Foran, T. G. , & Weber, M. M. (1939). An experimental study of the relation of homework to achievement in arithmetic. The Mathematics Teacher, 32(5), 212–214. 10.5951/MT.32.5.0212

[cl21431-bib-0004] Foyle, H. (1990). *Homework and cooperative learning: A classroom field experiment*. Emporia State University. https://files.eric.ed.gov/fulltext/ED350285.pdf

[cl21431-bib-0005] Gray, R. F. , & Allison, D. E. (1971). An experimental study of the relationship of homework to pupil success in computation with fractions. School Science and Mathematics, 71(4), 339–346. 10.1111/j.1949-8594.1971.tb15466.x

[cl21431-bib-0006] Koch, Jr., E. A. (1965). Homework in arithmetic. The Arithmetic Teacher, 12(1), 9–13. http://www.jstor.org/stable/41185071

[cl21431-bib-0007] Maertens, N. (1969). An analysis of the effects of arithmetic homework upon the arithmetic achievement of third‐grade pupils. The Arithmetic Teacher, 16(5), 383–389. 10.5951/AT.16.5.0383

[cl21431-bib-0008] Maertens, N. , & Johnston, J. (1972). Effects of arithmetic homework upon the attitudes and achievement of fourth, fifth and sixth grade pupils. School Science and Mathematics, 72(2), 117–126. 10.1111/j.1949-8594.1972.tb08815.x

[cl21431-bib-0009] McGrath, J. B. (1992). *Student and parental homework practices and the effect of English homework on student test scores* (Publication Number 9231359) [PhD, United States International University]. ProQuest Dissertations & Theses Global. https://queens.ezp1.qub.ac.uk/login?url=https://www.proquest.com/dissertations-theses/student-parental-homework-practices-effect/docview/304033702/se-2?accountid=13374

[cl21431-bib-0010] Townsend, S. (1995). *The effects of vocabulary homework on third grade achievement*. Kean College of New Jersey. https://files.eric.ed.gov/fulltext/ED379643.pdf

[cl21431-bib-0011] Tupesis, J. A. (1972). *Mathematics learning as a consequence of the learner's involvement in interactive problem‐solving tasks* (Publication Number 7309295) [PhD, The University of Wisconsin – Madison]. ProQuest Dissertations & Theses Global. https://queens.ezp1.qub.ac.uk/login?url=https://www.proquest.com/dissertations-theses/mathematics-learning-as-consequence-learners/docview/302608557/se-2?accountid=13374

[cl21431-bib-0012] Al‐Naqbi, A. K. (2014). The effects of instructional homework technique on chemistry achievement of the United Arab Emirates male and female tenth graders. International Journal for Research in Education, 35, 1–27. https://www.researchgate.net/publication/265602747_The_Effects_of_Instructional_Homework_Technique_on_Chemistry_Achievement_of_the_United_Arab_Emirates_Male_and_Female_Tenth_Graders

[cl21431-bib-0013] Bailey, L. B. (2006). Interactive homework: A tool for fostering parent–child interactions and improving learning outcomes for at‐risk young children. Early Childhood Education Journal, 34(2), 155–167. 10.1007/s10643-006-0114-y

[cl21431-bib-0014] Bartelet, D. , Ghysels, J. , Groot, W. , Haelermans, C. , & Maassen van den Brink, H. (2016). The differential effect of basic mathematics skills homework via a web‐based intelligent tutoring system across achievement subgroups and mathematics domains: A randomized field experiment. Journal of Educational Psychology, 108(1), 1–20. 10.1037/edu0000051

[cl21431-bib-0015] Bell, R. M. (2015). *An investigation of the impact of a flipped classroom instructional approach on high school students' content knowledge and attitudes toward the learning environment*. Brigham Young University. https://scholarsarchive.byu.edu/cgi/viewcontent.cgi?article=5443&context=etd

[cl21431-bib-0016] Chao, C.‐Y. , Chen, Y.‐T. , & Chuang, K.‐Y. (2015). Exploring students' learning attitude and achievement in flipped learning supported computer aided design curriculum: A study in high school engineering education. Computer Applications in Engineering Education, 23(4), 514–526. 10.1002/cae.21622

[cl21431-bib-0017] Clark, K. R. (2013). *Examining the effects of the flipped model of instruction on student engagement and performance in the secondary mathematics classroom: An action research study* (Publication Number 3592584) [D.Ed., Capella University]. ProQuest Dissertations & Theses Global. https://queens.ezp1.qub.ac.uk/login?url=https://www.proquest.com/dissertations-theses/examining-effects-flipped-model-instruction-on/docview/1437012328/se-2?accountid=13374

[cl21431-bib-0018] Dadas, J. E. (1976). *A study of the effects of assigning spiral exploratory homework upon achievement in and attitude towards mathematics* (Publication Number 7705344) [Educat.D., New York University]. ProQuest Dissertations & Theses Global. https://queens.ezp1.qub.ac.uk/login?url=https://www.proquest.com/dissertations-theses/study-effects-assigning-spiral-exploratory/docview/302796830/se-2?accountid=13374

[cl21431-bib-0019] Davis, J. M. (2004). *The impact of parental involvement: A study of the relationship between homework and kindergarten Texas Primary Reading Inventory scores* (Publication Number 3132081) [PhD, Texas A&M University]. ProQuest Dissertations & Theses Global. https://queens.ezp1.qub.ac.uk/login?url=https://www.proquest.com/dissertations-theses/impact-parental-involvement-study-relationship/docview/305074502/se-2?accountid=13374

[cl21431-bib-0020] Duffy, C. M. (2016). *The impact of flipped learning on student achievement in an eighth grade earth science classroom* (Publication Number 10126797) [Ed.D., Wilkes University]. ProQuest Dissertations & Theses Global. https://queens.ezp1.qub.ac.uk/login?url=https://www.proquest.com/dissertations-theses/impact-flipped-learning-on-student-achievement/docview/1808509112/se-2?accountid=13374

[cl21431-bib-0021] Esperanza, P. , Fabian, K. , & Toto, C. (2016). Flipped classroom model: Effects on performance, attitudes and perceptions in high school algebra. Adaptive and Adaptable Learning.

[cl21431-bib-0022] Flansburg, N. (2016). *Flipped learning instruction: Differentiating mathematics instruction through the use of technology* [Ed.D., Bethel University]. https://cdm16120.contentdm.oclc.org/digital/collection/p16120coll4/id/1089

[cl21431-bib-0023] Flick, A. (2019). *The effects of flipped learning in the sixth‐grade mathematics classroom* (Publication Number 13813313) [Ed.D., Missouri Baptist University] ProQuest Dissertations & Theses Global; Publicly Available Content Database. https://queens.ezp1.qub.ac.uk/login?url=https://www.proquest.com/dissertations-theses/effects-flipped-learning-sixth-grade-mathematics/docview/2204915840/se-2?accountid=13374

[cl21431-bib-0024] Foyle, H. C. (1984). *The effects of preparation and practice homework on student achievement in tenth‐grade American history* (Publication Number 8426314) [PhD, Kansas State University]. ProQuest Dissertations & Theses Global. https://queens.ezp1.qub.ac.uk/login?url=https://www.proquest.com/dissertations-theses/effects-preparation-practice-homework-on-student/docview/303330159/se-2?accountid=13374

[cl21431-bib-0025] Freet, B. A. (2016). *Flipping the classroom: An exploration of the effect of inverted learning on student achievement in a high school mathematics classroom*. Wheaton College Graduate School. https://files.library.wheaton.edu/etd/2016-MAT-EDU-FreetAllison.pdf

[cl21431-bib-0026] Glynn, J. (2013). *The effects of a flipped classroom on achievement and student attitudes in secondary chemistry*. Montana State University. https://scholarworks.montana.edu/xmlui/bitstream/handle/1/2882/GlynnJ0813.pdf?sequence=1

[cl21431-bib-0027] Howell, D. (2013). *Effects of an inverted instructional delivery model on achievement of ninth‐grade physical science honors students* (Publication Number 3602764) [Ed.D., Gardner‐Webb University]. ProQuest Dissertations & Theses Global. https://queens.ezp1.qub.ac.uk/login?url=https://www.proquest.com/dissertations-theses/effects-inverted-instructional-delivery-model-on/docview/1468701492/se-2?accountid=13374

[cl21431-bib-0028] Hungi, N. , & Ngware, M. (2017). Investigating the effects of community‐based interventions on mathematics achievement of girls from low‐income households in Kenya. Cogent Education, 4(1), 1290334. 10.1080/2331186X.2017.1290334

[cl21431-bib-0029] Kiesner, J. W. (1997). *The effects of a parental homework monitoring intervention on school engagement of high‐risk middle school students* (Publication Number 9723303) [PhD, University of Oregon]. ProQuest Dissertations & Theses Global. https://queens.ezp1.qub.ac.uk/login?url=https://www.proquest.com/dissertations-theses/effects-parental-homework-monitoring-intervention/docview/304384327/se-2?accountid=13374

[cl21431-bib-0030] Kırmızı, Ö. , & Kömeç, F. (2019). The impact of the flipped classroom on receptive and productive vocabulary learning. Dil ve Dilbilimi Çalışmaları Dergisi, 15(2), 437–449. 10.17263/jlls.586096

[cl21431-bib-0031] Kirvan, R. , Rakes, C. R. , & Zamora, R. (2015). Flipping an algebra classroom: Analyzing, modeling, and solving systems of linear equations. Computers in the Schools, 32(3–4), 201–223. 10.1080/07380569.2015.1093902

[cl21431-bib-0032] Lai, T.‐L. , Lin, F. T. , & Yueh, H.‐P. (2020). The effectiveness of team‐based flipped learning on a vocational high school economics classroom. Interactive Learning Environments, 28(1), 130–141. 10.1080/10494820.2018.1528284

[cl21431-bib-0033] Lonigan, C. J. , & Whitehurst, G. J. (1998). Relative efficacy of parent and teacher involvement in a shared‐reading intervention for preschool children from low‐income backgrounds. Early Childhood Research Quarterly, 13(2), 263–290. 10.1016/S0885-2006(99)80038-6

[cl21431-bib-0034] Meloy, L. L. (1987). *Effects of homework on language arts achievement in third and fourth‐grades* (Publication Number 8810173) [PhD, The University of Iowa]. ProQuest Dissertations & Theses Global. https://queens.ezp1.qub.ac.uk/login?url=https://www.proquest.com/dissertations-theses/effects-homework-on-language-arts-achievement/docview/303599752/se-2?accountid=13374

[cl21431-bib-0035] Metcalf, D. (2015). *The impact of flipping a middle school classroom on student achievement*. Califonia State University. https://scholarworks.calstate.edu/downloads/0k225b997

[cl21431-bib-0036] Montgomery, J. (2015). *The effects of flipped learning on middle school students' achievement with common core mathematics*. Califonia State University. https://scholarworks.calstate.edu/downloads/sq87bv07v

[cl21431-bib-0037] Nordstrom, H. M. (2012). The impact of online and traditional homework on the attitudes, achievement, and learning styles of sixth grade language arts students (Publication Number 3519068) [Ed.D., Trevecca Nazarene University]. ProQuest Dissertations & Theses Global. https://queens.ezp1.qub.ac.uk/login?url=https://www.proquest.com/dissertations-theses/impact-online-traditional-homework-on-attitudes/docview/1036615393/se-2?accountid=13374

[cl21431-bib-0038] Özcan, Z. Ç. , & Erktin, E. (2015). Enhancing mathematics achievement of elementary school students through homework assignments enriched with metacognitive questions. Eurasia Journal of Mathematics, Science & Technology Education, 11(6), 1415–1427. 10.12973/eurasia.2015.1402a

[cl21431-bib-0039] Ramaglia, H. (2015). The flipped mathematics classroom: A mixed methods study examining achievement, active learning, and perception (Publication Number 10002822) [PhD, Kansas State University]. ProQuest Dissertations & Theses Global. https://queens.ezp1.qub.ac.uk/login?url=https://www.proquest.com/dissertations-theses/flipped-mathematics-classroom-mixed-methods-study/docview/1761168648/se-2?accountid=13374

[cl21431-bib-0040] Ripley, D. (2015). *An examination of flipped instructional method on sixth graders' mathematics learning: Utilizing propensity score matching*. University of Nevada. https://scholarworks.unr.edu/bitstream/handle/11714/2518/Ripley_unr_0139D_11805.pdf?sequence=1&isAllowed=y

[cl21431-bib-0041] Robledo‐Ramón, P. , & García‐Sánchez, J.‐N. (2013). Strategy instruction for writing composition at school and at home. Studies in Psychology, 34(2), 161–174. 10.1174/021093913806751438

[cl21431-bib-0042] Saunders, J. M. (2014). The flipped classroom: Its effect on student academic achievement and critical thinking skills in high school mathematics (Publication Number 3645482) [Ed.D., Liberty University]. ProQuest Dissertations & Theses Global. https://queens.ezp1.qub.ac.uk/login?url=https://www.proquest.com/dissertations-theses/flipped-classroom-effect-on-student-academic/docview/1639087375/se-2?accountid=13374

[cl21431-bib-0043] Schultz, D. , Duffield, S. , Rasmussen, S. C. , & Wageman, J. (2014). Effects of the flipped classroom model on student performance for advanced placement high school chemistry students. Journal of Chemical Education, 91(9), 1334–1339. 10.1021/ed400868x

[cl21431-bib-0044] Schwankl, E. R. (2013). *Flipped classroom: Effects on achievement and student perception* (Publication Number 1523826) [M.S.E., Southwest Minnesota State University]. ProQuest Dissertations & Theses Global. https://queens.ezp1.qub.ac.uk/login?url=https://www.proquest.com/dissertations-theses/flipped-classroom-effects-on-achievement-student/docview/1441947201/se-2?accountid=13374

[cl21431-bib-0045] Smith, J. P. (2015). *The efficacy of a flipped learning classroom* (Publication Number 3719573) [Ed.D., McKendree University]. ProQuest Dissertations & Theses Global. https://queens.ezp1.qub.ac.uk/login?url=https://www.proquest.com/dissertations-theses/efficacy-flipped-learning-classroom/docview/1713692218/se-2?accountid=13374

[cl21431-bib-0046] Špilka, R. (2014, November 18‐20). *Pedagogical experiment with online visualization of mathematical models in math teaching on elementary school*. The 7th International Conference of Education, Research and Innovation, Seville, Spain.

[cl21431-bib-0047] Tamayo, L. F. (1992). *Hispanic parent monitoring of seventh‐grade mathematics homework assignments and relationship with achievement and self‐esteem* (Publication Number 9233167) [PhD, University of Massachusetts Amherst]. ProQuest Dissertations & Theses Global. https://queens.ezp1.qub.ac.uk/login?url=https://www.proquest.com/dissertations-theses/hispanic-parent-monitoring-seventh-grade/docview/303993007/se-2?accountid=13374

[cl21431-bib-0048] Tsai, C.‐W. , Shen, P.‐D. , & Lu, Y.‐J. (2015). The effects of problem‐based learning with flipped classroom on elementary students' computing skills: A case study of the production of ebooks. International Journal of Information and Communication Technology Education, 11(2), 32–40. 10.4018/ijicte.2015040103

[cl21431-bib-0049] Van Voorhis, F. L. (2010). Adding families to the homework equation: A longitudinal study of mathematics achievement. Education and Urban Society, 43(3), 313–338. 10.1177/0013124510380236

[cl21431-bib-0050] Wiginton, B. L. (2013). Flipped instruction: An investigation into the effect of learning environment on student self‐efficacy, learning style, and academic achievement in an Algebra I classroom (Publication Number 3612166) [PhD, The University of Alabama]. ProQuest Dissertations & Theses Global. https://queens.ezp1.qub.ac.uk/login?url=https://www.proquest.com/dissertations-theses/flipped-instruction-investigation-into-effect/docview/1505373684/se-2?accountid=13374

[cl21431-bib-0051] Yousefzadeh, M. (2015). The effect of flipped learning (revised learning) on Iranian students' learning outcomes. Advances in Language and Literary Studies, 6(5), 209–213. 10.7575/aiac.alls.v.6n.5p.209

[cl21431-bib-0052] Ackerman, P. L. , Chamorro‐Premuzic, T. , & Furnham, A. (2011). Trait complexes and academic achievement: Old and new ways of examining personality in educational contexts. British Journal of Educational Psychology, 81(Pt. 1), 27–40. 10.1348/000709910x522564 21391962

[cl21431-bib-0053] de Araujo, Z. , Otten, S. , & Birisci, S. (2017). Conceptualizing “homework” in flipped mathematics classes. Journal of Educational Technology & Society, 20(1), 248–260. https://www.researchgate.net/publication/312056326_Conceptualizing_Homework_in_Flipped_Mathematics_Classes

[cl21431-bib-0054] Baş, G. , Senturk, C. , & Ciğerci, F. M. (2017). Homework and academic achievement: A meta‐analytic review of research. Issues in Educational Research, 27, 31–50. https://www.researchgate.net/publication/312372750_Homework_and_academic_achievement_A_meta-analytic_review_of_research

[cl21431-bib-0055] Becker, H. J. , & Epstein, J. L. (1982). Parent involvement: A survey of teacher practices. The Elementary School Journal, 83(2), 85–102. 10.1086/461297

[cl21431-bib-0056] Blazer, C. (2009). *Homework literature review*. https://files.eric.ed.gov/fulltext/ED536245.pdf

[cl21431-bib-0057] Borenstein, M. , Hedges, L. V. , Higgins, J. P. T. , & Rothstein, H. R. (Eds.). (2009). Identifying and quantifying heterogeneity. In Introduction to meta‐analysis (pp. 107–125). John Wiley & Sons, Ltd. 10.1002/9780470743386.ch16

[cl21431-bib-0058] Cadime, I. , Cruz, J. , Silva, C. , & Ribeiro, I. (2017). Homework self‐regulation strategies: A gender and educational‐level invariance analysis. Psicologia: Reflexão e Crítica, 30(1), 8. 10.1186/s41155-017-0062-z PMC6966999 32026986

[cl21431-bib-0059] Callahan, J. T. (2016). Assessing online homework in first‐semester calculus. PRIMUS, 26(6), 545–556. 10.1080/10511970.2015.1128501

[cl21431-bib-0060] Cheema, J. R. , & Sheridan, K. M. (2015). Time spent on homework, mathematics anxiety and mathematics achievement: Evidence from a US sample. Issues in Educational Research, 25, 246–259. https://www.researchgate.net/publication/282757990_Time_spent_on_homework_mathematics_anxiety_and_mathematics_achievement_Evidence_from_a_US_sample

[cl21431-bib-0061] Cooper, H. (1989). Homework. Longman.

[cl21431-bib-0062] Cooper, H. , & Nye, B. (1994). Homework for students with learning disabilities: The implications of research for policy and practice. Journal of Learning Disabilities, 27(8), 470–479. 10.1177/002221949402700802 7989851

[cl21431-bib-0063] Cooper, H. , Robinson, J. C. , & Patall, E. A. (2006). Does homework improve academic achievement? A synthesis of research, 1987–2003. Review of Educational Research, 76(1), 1–62. 10.3102/00346543076001001

[cl21431-bib-0064] Costley, K. C. (2013). *Does homework really improve achievement*. Arkansas Tech University. https://files.eric.ed.gov/fulltext/ED542436.pdf

[cl21431-bib-0065] Deysolong, J. (2023). *Investigating the effects of homework on student learning and academic performance*. Gusa Regional Science High School‐X. 10.6084/m9.figshare.23002418.v3

[cl21431-bib-0066] Epstein, J. L. , & Van Voorhis, F. L. (2001). More than minutes: Teachers' roles in designing homework. Educational Psychologist, 36(3), 181–193. 10.1207/S15326985EP3603_4

[cl21431-bib-0067] Fan, H. , Xu, J. , Cai, Z. , He, J. , & Fan, X. (2017). Homework and students' achievement in math and science: A 30‐year meta‐analysis, 1986–2015. Educational Research Review, 20, 35–54. 10.1016/j.edurev.2016.11.003

[cl21431-bib-0068] Federick, A. (2020). Finland education system. International Journal of Science and Society, 2(2), 21–32. 10.54783/ijsoc.v2i2.88

[cl21431-bib-0069] Fernández‐Alonso, R. , Suárez‐Álvarez, J. , & Muñiz, J. (2015). Adolescents' homework performance in mathematics and science: Personal factors and teaching practices. Journal of Educational Psychology, 107, 1075–1085. 10.1037/edu0000032

[cl21431-bib-0070] Frog Education . (2019). *The power of a good homework policy*. Retrieved February 26, 2024, from https://inspire.frogeducation.com/news/homeworkpolicy

[cl21431-bib-0071] Gill, B. P. , & Schlossman, S. L. (2003). A nation at rest: The American way of homework [Academic Learning & Achievement 3550]. Educational Evaluation and Policy Analysis, 25(3), 319–337. 10.3102/01623737025003319

[cl21431-bib-0072] Gill, B. P. , & Schlossman, S. L. (2004). Villain or savior? The American discourse on homework, 1850–2003. Theory Into Practice, 43(3), 174–181. 10.1207/s15430421tip4303_2

[cl21431-bib-0073] González, N. , Andrade, R. , Civil, M. , & Moll, L. (2001). Bridging funds of distributed knowledge: Creating zones of practices in mathematics. Journal of Education for Students Placed at Risk (JESPAR), 6(1–2), 115–132. 10.1207/S15327671ESPR0601-2_7

[cl21431-bib-0074] Guo, L. , Li, J. , Xu, Z. , Hu, X. , Liu, C. , Xing, X. , Li, X. , White, H. , & Yang, K. (2021). PROTOCOL: The relationship between homework time and academic performance among K‐12 students: A systematic review. Campbell Systematic Reviews, 17(4), e1199. 10.1002/cl2.1199 36950338 PMC8988773

[cl21431-bib-0075] Güven, U. , & Akçay, A. O. (2019). Trends of homework in mathematics: Comparative research based on TIMSS study. International Journal of Instruction, 12, 1367–1382. 10.29333/iji.2019.12187a

[cl21431-bib-0076] Hallam, S. (2004). *Homework: The evidence*. University of London.

[cl21431-bib-0077] Higgins, J. P. T. , & Thompson, S. G. (2002). Quantifying heterogeneity in a meta‐analysis. Statistics in Medicine, 21(11), 1539–1558. 10.1002/sim.1186 12111919

[cl21431-bib-0078] Higgins, J. P. T. , & Green, S. (Eds.). (2011). *Cochrane handbook for systematic reviews of interventions version 5.1.0* [updated March 2011]. The Cochrane Collaboration. http://handbook-5-1.cochrane.org/

[cl21431-bib-0079] Jerrim, J. , Lopez‐Agudo, L. A. , & Marcenaro‐Gutierrez, O. D. (2019). The relationship between homework and the academic progress of children in Spain during compulsory elementary education: A twin fixed‐effects approach. British Educational Research Journal, 45(5), 1021–1049. 10.1002/berj.3549

[cl21431-bib-0080] Keith, T. Z. (1982). Time spent on homework and high school grades: A large‐sample path analysis. Journal of Educational Psychology, 74, 248–253. 10.1037/0022-0663.74.2.248

[cl21431-bib-0081] Kinyua, K. , & Odiemo, L. (2018). Validity and reliability of teacher‐made tests: Case study of year 11 physics in Nyahururu District of Kenya.

[cl21431-bib-0082] Kitsantas, A. , Cheema, J. , & Ware, H. W. (2011). Mathematics achievement: The role of homework and self‐efficacy beliefs. Journal of Advanced Academics, 22(2), 310–339. 10.1177/1932202x1102200206

[cl21431-bib-0083] Kralovec, E. , & Buell, J. (2000). The end of homework: How homework disrupts families, overburdens children, and limits learning. Beacon Press.

[cl21431-bib-0084] Krejtz, K. , Duchowski, A. T. , Niedzielska, A. , Biele, C. , & Krejtz, I. (2018). Eye tracking cognitive load using pupil diameter and microsaccades with fixed gaze. PLoS One, 13(9), e0203629. 10.1371/journal.pone.0203629 30216385 PMC6138399

[cl21431-bib-0085] Lee, J. F. , & Pruitt, K. W. (1979). Homework assignments: Classroom games or teaching tools? The Clearing House: A Journal of Educational Strategies, Issues and Ideas, 53(1), 31–35. 10.1080/00098655.1979.9957112

[cl21431-bib-0086] Lucas, A. R. (2012). Using WeBWorK, a web‐based homework delivery and grading system, to help prepare students for active learning. PRIMUS, 22(2), 97–107. 10.1080/10511970.2010.497834

[cl21431-bib-0087] Mae Gambong Luengas, R. , & Deloy, E. D. A. (2022). Delving into the perspectives of teachers in no homework policy: A qualitative investigation. International Journal of Research Publications, 110(1), 428–456. 10.47119/IJRP10011011020223961

[cl21431-bib-0088] Mendicino, M. , Razzaq, L. , & Heffernan, N. T. (2009). A comparison of traditional homework to computer‐supported homework. Journal of Research on Technology in Education, 41(3), 331–359. 10.1080/15391523.2009.10782534

[cl21431-bib-0089] Ministry of Education of the People's Republic of China . (2021). *Strengthening the management of homework in compulsory education schools*. https://www.gov.cn/zhengce/zhengceku/2021-04/25/content_5602131.htm

[cl21431-bib-0090] Moher, D. , Liberati, A. , Tetzlaff, J. , & Altman, D. G. (2009). Preferred reporting items for systematic reviews and meta‐analyses: The PRISMA statement. PLoS Medicine, 6(7), e1000097. 10.1371/journal.pmed.1000097 19621072 PMC2707599

[cl21431-bib-0091] Muhammad, H. I. (2015). Thorndike theory and it's application in learning. At‐Ta'lim: Jurnal Pendidikan, 1(1), 37–47. https://ejournal.inzah.ac.id/index.php/attalim/article/view/166

[cl21431-bib-0092] Muhlenbruck, L. , Cooper, H. , Nye, B. , & Lindsay, J. J. (1999). Homework and achievement: Explaining the different strengths of relation at the elementary and secondary school levels. Social Psychology of Education, 3(4), 295–317. 10.1023/A:1009680513901

[cl21431-bib-0093] Qiao, L. , & Fan, H. (2020). The learning effectiveness difference between web‐based homework and traditional homework: A meta‐analysis [网络家庭作用与传统家庭作业学习效果差异元分析]. Open Education Research, 26(1), 100–110. 10.13966/j.cnki.kfjyyj.2020.01.011

[cl21431-bib-0094] Reteig, L. C. , van den Brink, R. L. , Prinssen, S. , Cohen, M. X. , & Slagter, H. A. (2019). Sustaining attention for a prolonged period of time increases temporal variability in cortical responses. Cortex, 117, 16–32. 10.1016/j.cortex.2019.02.016 30925309

[cl21431-bib-1000] Roderique, T. W. , Polloway, E. A. , Cumblad, C. , Epstein, M. H. , & Bursuck, W. D. (1994). Homework: A survey of policies in the united states. Journal of Learning Disabilities, 27(8), 481–487. 7989852 10.1177/002221949402700803

[cl21431-bib-0095] Setiabudi, A. , Mulyadi, M. , & Puspita, H. (2019). An analysis of validity and reliability of a teacher‐made test. Journal of English Education and Teaching, 3, 522–532. 10.33369/jeet.3.4.522-532

[cl21431-bib-0096] South View Primary School . (2024). *School home partnership: Homework policy*. Ministry of Education of Singapore. Retrieved February 26, 2024, from https://www.southviewpri.moe.edu.sg/info-hub/school-home-partnership-homework-policy/

[cl21431-bib-0097] Sterne, J. A. , Hernán, M. A. , Reeves, B. C. , Savović, J. , Berkman, N. D. , Viswanathan, M. , Henry, D. , Altman, D. G. , Ansari, M. T. , Boutron, I. , Carpenter, J. R. , Chan, A.‐W. , Churchill, R. , Deeks, J. J. , Hróbjartsson, A. , Kirkham, J. , Jüni, P. , Loke, Y. K. , Pigott, T. D. , … Higgins, J. P. (2016). ROBINS‐I: A tool for assessing risk of bias in non‐randomised studies of interventions. BMJ, 355, 4919. 10.1136/bmj.i4919 PMC5062054 27733354

[cl21431-bib-0098] Trautwein, U. (2003). The relationship between homework and achievement—Still much of a mystery. Educational Psychology Review, 15(2), 115–145. 10.1023/A:1023460414243

[cl21431-bib-0099] Trautwein, U. , & Lüdtke, O. (2007). Students' self‐reported effort and time on homework in six school subjects: Between‐students differences and within‐student variation. Journal of Educational Psychology, 99(2), 432–444. 10.1037/0022-0663.99.2.432

[cl21431-bib-0100] Twenge, J. M. , Zhang, L. , & Im, C. (2004). It's beyond my control: A cross‐temporal meta‐analysis of increasing externality in locus of control, 1960–2002. Personality and Social Psychology Review, 8(3), 308–319. 10.1207/s15327957pspr0803_5 15454351

[cl21431-bib-0101] Van Voorhis, F. L. (2003). Interactive homework in middle school: Effects on family involvement and science achievement. The Journal of Educational Research, 96(6), 323–338. 10.1080/00220670309596616

[cl21431-bib-0102] Waddington, H. , Aloe, A. M. , Becker, B. J. , Djimeu, E. W. , Hombrados, J. G. , Tugwell, P. , Wells, G. , & Reeves, B. (2017). Quasi‐experimental study designs series‐paper 6: Risk of bias assessment. Journal of Clinical Epidemiology, 89, 43–52. 10.1016/j.jclinepi.2017.02.015 28351693

[cl21431-bib-0103] World Health Organization Regional Office for Europe . (2016). *Growing up unequal: Gender and socioeconomic differences in young people's health and well‐being*. World Health Organization. https://www.who.int/europe/publications/i/item/9789289051361

[cl21431-bib-0104] Xu, J. (2009). School location, student achievement, and homework management reported by middle school students. The School Community Journal, 19(2), 27–43.

[cl21431-bib-0105] Xue, H. , & Zhang, Y. (2019). Analysis of the academic burden level and difference of junior high school students in China: An empirical study based on CEPS2015 data. Journal of Capital Normal University (Social Sciences Edition), 1(05), 147–166. https://kns.cnki.net/kcms/detail/detail.aspx?dbcode=CJFD&dbname=CJFDLAST2019&filename=SDSD201905018&uniplatform=NZKPT&v=W09bfInSi3TFZzGrpMhZgTS6W2LFmcDrerLxlHBZx1rnUytv0l9DAIczVXTHao0N

[cl21431-bib-0106] Zhu, Y. (2015). Homework and mathematics learning: What can we learn from the TIMSS series studies in the last two decades? In J. A. Middleton , J. Cai , & S. Hwang (Eds.), Large‐scale studies in mathematics education (pp. 209–234). Springer International Publishing. 10.1007/978-3-319-07716-1_10

